# Niosome-mediated melatonin delivery via SilMA/GelMA microneedles promotes healing of sleep deprivation-aggravated oral ulcers

**DOI:** 10.1093/rb/rbag180

**Published:** 2026-08-18

**Authors:** Ying Xie, Qi Huang, Xinzhi Jiang, Kehao Liu, Kamoran Tuerhong, Chengji Shi, Renjie Tang, Xuehui Hu, Youchun Zhang, Xiang Li, Mingcong Yang, Yuting He, Haixia Dang, Ming Wang, Danfeng Shen, Sheng Yang, Peng Yu, Yuzhou Li

**Affiliations:** Department of Prosthodontics, The Affiliated Stomatological Hospital of Chongqing Medical-University, Chongqing 401147, China; Department of Prosthodontics, The Affiliated Stomatological Hospital of Chongqing Medical-University, Chongqing 401147, China; Department of Prosthodontics, The Affiliated Stomatological Hospital of Chongqing Medical-University, Chongqing 401147, China; Department of Prosthodontics, The Affiliated Stomatological Hospital of Chongqing Medical-University, Chongqing 401147, China; Department of Prosthodontics, The Affiliated Stomatological Hospital of Chongqing Medical-University, Chongqing 401147, China; Department of Prosthodontics, The Affiliated Stomatological Hospital of Chongqing Medical-University, Chongqing 401147, China; Department of Prosthodontics, The Affiliated Stomatological Hospital of Chongqing Medical-University, Chongqing 401147, China; Department of Orthodontics, The Affiliated Stomatological Hospital of Chongqing Medical-University, Chongqing 401147, China; Department of Prosthodontics, The Affiliated Stomatological Hospital of Chongqing Medical-University, Chongqing 401147, China; Department of Prosthodontics, The Affiliated Stomatological Hospital of Chongqing Medical-University, Chongqing 401147, China; Department of Periodontology, The Affiliated Stomatological Hospital of Chongqing Medical-University, Chongqing 401147, China; Department of Prosthodontics, The Affiliated Stomatological Hospital of Chongqing Medical-University, Chongqing 401147, China; Department of Prosthodontics, The Affiliated Stomatological Hospital of Chongqing Medical-University, Chongqing 401147, China; Department of Prosthodontics, The Affiliated Stomatological Hospital of Chongqing Medical-University, Chongqing 401147, China; Department of Prosthodontics, The Affiliated Stomatological Hospital of Chongqing Medical-University, Chongqing 401147, China; Department of Prosthodontics, The Affiliated Stomatological Hospital of Chongqing Medical-University, Chongqing 401147, China; Department of Cariology and Endodontology, Peking University School and Hospital of Stomatology & National Center for Stomatology & National Clinical Research Center for Oral Diseases & National Engineering Research Center of Oral Biomaterials and Digital Medical Devices. Beijing 100081, China; Department of Prosthodontics, The Affiliated Stomatological Hospital of Chongqing Medical-University, Chongqing 401147, China; Chongqing Key Laboratory of Oral Diseases & Chongqing Municipal Key Laboratory of Oral Biomedical Engineering of Higher Education & Chongqing Municipal Health Commission Key Laboratory of Oral Biomedical Engineering, Chongqing 401147, China

**Keywords:** melatonin, noisome, neutrophil extracellular traps, sleep deprivation, oral ulcer

## Abstract

Sleep disturbance disrupts immune homeostasis and impairs oral mucosal healing, yet effective local immunomodulatory strategies remain limited. Here, we identify excessive neutrophil extracellular traps (NETs) as a key mediator linking sleep deprivation to delayed oral wound closure. NETs-associated proteases compromise epithelial barrier function and suppress repair cell migration. To address this, we developed a melatonin-loaded niosome-incorporated silk fibroin methacrylate (SilMA)/gelatin methacryloyl (GelMA) composite hydrogel microneedle (Nio-MT@S-G MN) for localized melatonin delivery and regulation of the NET-associated inflammatory microenvironment. Niosomes achieve efficient encapsulation and sustained melatonin release, while SilMA/GelMA microneedles enable transmucosal administration with favorable mechanical strength. *In vitro*, Nio-MT@S-G MN promotes keratinocyte migration, reduces NETs marker expression and extracellular DNA release in neutrophils, and alleviates NETs-mediated inhibition of tight junction Zonula occludens-1 (ZO-1) expression. In mice combining sleep deprivation and oral ulcers, local Nio-MT@S-G MN application attenuates NETs deposition and inflammation, restores epithelial barrier proteins and significantly accelerates ulcer closure. By integrating controlled drug delivery with immune microenvironment regulation, this microneedle provides a translational strategy for treating sleep disorder-aggravated mucosal injuries. It may also be applicable to other chronic wounds characterized by dysregulated neutrophil responses.

## Graphical abstract

**Graphical Abstract:**
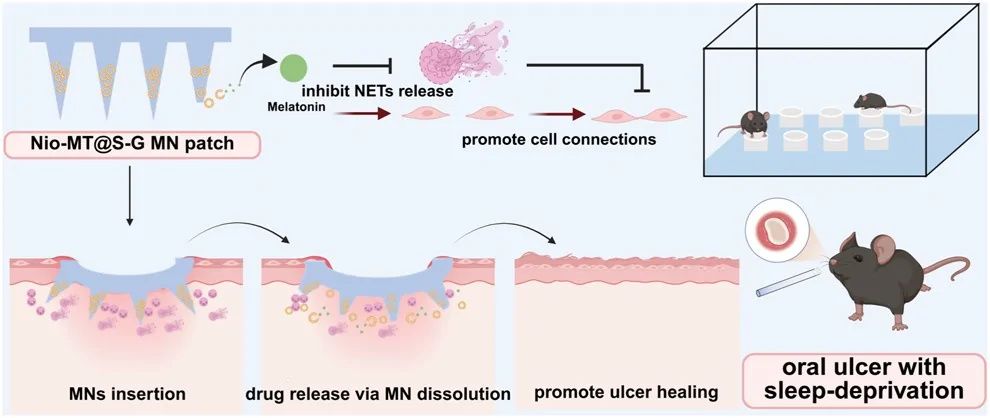

## Introduction

Oral ulcers are among the most common lesions of the oral mucosa and constitute a major cause of oral mucosal pain [1, 2]. Under normal physiological conditions, oral ulcers are often regarded as self-limiting despite their high incidence and considerable discomfort [3]. However, oral ulcers in the presence of sleep disturbance have been reported to exhibit delayed healing and aggravated inflammatory responses, resulting in a greater disease burden [4]. Sleep disorders are now recognized as a major global health challenge and highly prevalent among adults, largely driven by chronic psychosocial stress and modern lifestyle pressures [5–7]. Emerging evidence suggests that sleep disturbance markedly affects oral mucosal health and is associated with multiple oral inflammatory conditions, including gingivitis, periodontitis and oral ulcers [8]. Notably, animal studies have demonstrated that sleep deprivation can significantly aggravate oral ulcer inflammation and delay the healing process [4]. Despite this evidence, therapeutic strategies specifically designed for oral ulcers under sleep-deprived conditions are still lacking.

Neutrophils are central to both oral mucosal inflammation and sleep-related immune dysregulation. As the most abundant innate immune cells, neutrophils serve as early responders during tissue injury, contributing to antimicrobial defense, inflammatory orchestration and wound repair through phagocytosis, degranulation, reactive oxygen species (ROS) release and neutrophil extracellular traps (NETs) formation [9–13]. However, excessive or persistent neutrophil activation can amplify tissue damage and impair healing [14, 15]. In the context of oral ulcer, neutrophils are central to mucosal immune homeostasis and have been increasingly implicated in oral inflammatory and ulcerative diseases [16]. In sleep-related disorders, neutrophils have emerged as important immune effectors because sleep deprivation is associated with alterations in neutrophil abundance and effector function, indicating that neutrophil dysregulation is closely involved in sleep-related inflammatory pathology [17]. Given that sleep deprivation has been reported to aggravate oral ulcer inflammation and delay healing, and that the underlying mechanism may involve neuro-immune-endocrine dysregulation [4, 18], it is important to consider how sleep loss shapes neutrophil biology beyond the peripheral circulation. Recent evidence indicates that sleep deprivation fundamentally reprograms the bone marrow, where neutrophils originate, to produce cells that are intrinsically primed for heightened inflammatory responses. McAlpine *et al.* [19] demonstrated that sleep fragmentation reduces hypothalamic hypocretin (orexin) production, which normally restricts CSF1 production by pre-neutrophils in the bone marrow. Sleep deprivation relieves this restraint, leading to elevated CSF1 levels that drive myeloid-biased hematopoiesis and the generation of neutrophils that are more responsive to subsequent inflammatory stimuli [19]. Consistent with this, sleep-deprived rats exhibit mild regenerative neutrophilia accompanied by immature neutrophils, and sleep-deprived medical residents show altered neutrophil function [17, 18]. Thus, neutrophils activated under sleep-deprived conditions may be particularly prone to release NETs at sites of tissue injury, contributing to aggravated inflammation and delayed healing in oral ulcers. This positions neutrophils and their effector functions-particularly NET formation-as rational therapeutic targets for sleep disturbance-associated oral mucosal pathologies.

At present, the treatment of oral ulcers mainly relies on topical anti-inflammatory, analgesic, barrier-protective and pro-epithelialization agents [20–22]. However, these approaches remain suboptimal for sleep deprivation-aggravated oral ulcers for three main reasons. First, drugs applied to the oral mucosa are readily diluted and cleared by saliva and mechanical activities, resulting in poor local retention and limited therapeutic persistence [23, 24]. Second, and most importantly, current oral mucosal therapies are not designed to provide long-acting and effective intervention against the neutrophil-dominant inflammatory state that characterizes sleep deprivation-aggravated oral ulcers. In this setting, treatment needs not only to achieve durable local retention in the oral ulcer, but also to effectively alleviate neutrophil-associated inflammatory injury, oxidative stress and delayed wound healing. Therefore, there remains an urgent need for a localized therapeutic system that enables sustained and effective transmucosal delivery while concurrently targeting neutrophil-associated pathology and supporting wound healing in this specific pathological setting.

To improve local drug delivery to the oral mucosa, researchers have explored a variety of material platforms, including mucoadhesive patches, hydrogels and microneedle systems [25–29]. Among them, hydrogel-based formulations can enhance local retention and mucosal contact, whereas microneedle-based systems enable more efficient transmucosal deposition and delivery of therapeutics at the lesion site [30, 31]. On this basis, nanoscale carriers such as liposomes, niosomes, ethosomes, transferosomes and other lipid-based nanoparticles have been further incorporated to protect labile drugs, improve loading efficiency, enhance local stability and achieve more sustained release [32–34]. Compared with single-level formulations, such hierarchical systems offer a distinct advantage by integrating macro-/micro-scale mucosal delivery with nanoscale drug encapsulation, thereby enabling sequential improvement in drug retention, protection and controlled release [35]. This multi-level delivery strategy is particularly attractive for oral ulcers under sleep-disordered conditions, where durable local exposure and prolonged therapeutic action are both required.

Therefore, we constructed the Nio-MT@S-G MN for localized transmucosal delivery and sustained release. This integrated system was designed to address the key limitations of current therapy in a coordinated manner: melatonin was selected for its well-established antioxidant and immunomodulatory properties, which are relevant to neutrophil-associated inflammatory pathology [36–41]; niosomal encapsulation improves melatonin stability and local retention, thereby reducing the disadvantage of rapid drug loss [42–45]; the Silk Fibroin Methacryloyl/Gelatin Methacryloyl (SilMA/GelMA) microneedle platform enables localized and sustained release, and a favorable microenvironment for epithelial repair [46]. In this study, we systematically evaluated the physicochemical properties, biosafety and therapeutic potential of Nio-MT@S-G MN in sleep deprivation-aggravated oral ulcers, with a particular focus on epithelial repair, NET-associated regulation and mucosal healing.

## Materials and methods

### Materials

Span 60 (Cat. No. S112961) was purchased from Aladdin (Shanghai, China). Cholesterol (Cat. No. C8280), dialysis bag (Cat. No. YA1071, 8–14 kDa), hematoxylin and eosin (HE) staining (Cat. No. G1120), Crystal violet (Cat. No. IC0600) and Mouse Bone Marrow Neutrophil Isolation Solution Kit (Cat. No. P8550) were purchased from Solarbio (Beijing, China). Melatonin (Cat. No. ST1497), an Alexa Fluor 555-conjugated donkey anti-rabbit secondary antibody (Cat. No. A0453; 1:300), DAPI (Cat. No. C1002; 1:1000), RIPA buffer containing protease inhibitors (Cat. No. P0013B) and BCA assay kit (Cat. No. P0011) were purchased from Beyotime Biotechnology Co., Ltd. (Shanghai, China). GelMA, SilMA and Lithium Phenyl-2,4,6-trimethylbenzoylphosphinate (LAP) were purchased from EFL (Suzhou, China). The Polydimethylsiloxane (PDMS) microneedle mold was purchased from Henan Winna Benton Biotechnology Co., Ltd. (Henan, China). Avertin anesthetic (Cat. No. DW3101) was purchased from Duowo (Shanghai, China). HaCaT cells were obtained from Hunan Fenghui Biotechnology Co., Ltd. (Hunan, China). Cell Counting Kit-8 (CCK8) (Cat. No. HY-K0301) and Cl-amidine hydrochloride (HY-100574A) were purchased from MedChemExpress (Shanghai, China). Calcein-acetoxymethyl ester /propidium iodide (PI, Cat. No. BB-4126) was purchased from BestBio (China). Ionomycin (Cat. No. I3909) was purchased from Sigma-Aldrich (Shanghai, China). A rabbit anti-zonula occludens-1 (ZO-1) primary antibody (Cat. No. 40-2200; 1:600), Power SYBR™ Green PCR Master Mix (Cat. No. 4368577) and SYTOX Green (Cat. No. S7020; 1:50 000) were purchased from Thermo Fisher Scientific (Shanghai, China). Citrullinated histone H3 (CitH3, Cat. No. ab5103; 1:1000), peptidylarginine deiminase 4 (PAD4, Cat. No. ab214810; 1:1000) and myeloperoxidase (MPO, Cat. No. ab208670, 1:1000) were purchased from Abcam (Cambridge, UK). TRIzol reagent and RNase-free water were purchased from Takara (Japan).

### Animals

All animal experiments were conducted in accordance with the Guide for the Care and Use of Laboratory Animals and were approved by the Medical Ethics Committee of the Affiliated Stomatology Hospital of Chongqing Medical University (Approval No. 2025(053)). Eight-week-old female C57BL/6 mice weighing 15 ± 5 g were purchased from the Experimental Animal Center of Chongqing Medical University.

### Mouse models

#### Oral ulcer induction

Mice were anesthetized by intraperitoneal injection of 0.3% sodium pentobarbital (0.15 mL per 10 g body weight, equivalent to 45 mg/kg) [47]. A cotton pellet (3 mm outer diameter, 2 mm inner diameter) soaked in 90% phenol was applied to the bilateral buccal mucosa for 45 s on each side to induce uniform ulcers [48].

#### Sleep deprivation

Sleep deprivation was induced using a modified multiple-platform method, a well-established paradigm that preferentially disrupts sleep while minimizing non-specific stress responses, thereby more closely mimicking clinical sleep disturbance rather than generalized stress. Mice were divided into three groups: oral ulcers under normal sleep conditions (OU), oral ulcers under sleep deprivation (OUS) and OUS+Nio-MT@S-G MN. Oral ulcers were induced on Day 1. Mice in the OUS and OUS+Nio-MT@S-G MN groups were subjected to sleep deprivation in a water chamber containing small platforms, where loss of muscle tone during sleep caused them to fall into the water and awaken. In contrast, mice in the OU group were maintained on dry platforms under otherwise comparable conditions. All mice were acclimated to the apparatus for 2 days before the formal experiment. On Day 2 after ulcer induction, sleep deprivation was initiated and continued simultaneously with Nio-MT@S-G MN treatment in the OUS+Nio-MT@S-G MN group until Day 6. Mice were euthanized by CO₂ asphyxiation on Day 6, and buccal mucosal tissues were collected for subsequent analyses.

#### Ulcer measurement

On Day 2 post-induction, ulcer areas were photographed under isoflurane anesthesia and measured using ImageJ. Daily monitoring continued under anesthesia, with healing rate calculated as:


$$\text{Healing}\;\text{Rate}\;(\%)\;=\;\frac{A_{t}-A_{2}}{A_{2}}\times 100\%\;(A_{2}:\mathrm{initial}\;\mathrm{area};\;A_{t}:\mathrm{area}\;\mathrm{at}\;\mathrm{time}\;\mathrm{point}).$$

### Preparation and characterization of melatonin-loaded niosomes

24 mg Span 60, 8 mg cholesterol (ratio 3:1) and melatonin of different quantities (4, 6, 8, 10 mg) were dissolved in 5 mL of chloroform. The mixture was incubated at 37°C for 10 min and sonicated for 10 min in a water bath sonicator (Kudos SK3210HP, China). The thin film was obtained by evaporating the mixture solution at 30°C for 30 min under 200 mbar pressure on a rotary evaporator (Yarong, China), followed by further drying under reduced pressure at room temperature for 12 h in a vacuum desiccator. Sterile deionized water (5 mL) was added to the thin film and sonicated until all of the thin films were dissolved. The blank niosome (Nio) was prepared according to these same processes except for the addition of melatonin. The melatonin-loaded niosomes (Nio-MT) were stored at 4°C for further use. Each sample was diluted with deionized water (1:30,00 v/v) and then analyzed using an SZ-100 nanoparticle size analyzer (Brookhaven, USA) to evaluate the particle size and zeta potential of Nio and Nio-MT (30 min). This procedure was performed in triplicate. The morphology of Nio and Nio-MT was characterized by using transmission electron microscopy (TEM, FEI Company, USA).

### Establishment of the melatonin standard curve

A fluorescence-based standard curve of melatonin was established prior to the determination of drug encapsulation and release. Briefly, melatonin was dissolved in methanol to prepare standard solutions at concentrations of 0, 0.4, 0.8, 1.2, 1.6, 2.0, 2.4, 2.8, 3.2, 3.6 and 4.0 μg mL^−1^. Then, 100 μL of each standard solution was added into a black 96-well plate in triplicate. To avoid photodegradation, the plate was wrapped in aluminum foil and protected from light. Fluorescence intensity was measured using a microplate reader (PerkinElmer, USA) at an excitation wavelength of 285 nm and an emission wavelength of 345 nm. The standard calibration curve was generated based on melatonin concentration and fluorescence intensity (Table S2, available as supplementary data at *Regenerative Biomaterials* online).

### Determination of encapsulation efficiency rate and drug loading rate

The encapsulation efficiency rate (EER) and drug loading rate (DLR) of melatonin in Nio-MT were determined by fluorescence quantification. Briefly, 1 mL of freshly prepared Nio-MT suspension was centrifuged at 16 000 g at 4 °C for 2 h to separate the encapsulated and unencapsulated melatonin. After centrifugation, the precipitated niosomal fraction was collected and dissolved in methanol to disrupt the vesicular structure and release the encapsulated melatonin. The fluorescence intensity of the resulting solution was measured using a microplate reader, and the melatonin concentration was determined according to the standard calibration curve.

The EER and DLR were calculated using the following equations:


$$\text{EER}\;(\%)=\frac{\text{mass}\;\text{of}\;\text{encapsulated}\;\text{melatonin}}{\text{mass}\;\text{of}\;\text{total}\;\text{melatonin}\;\text{added}}\times 100\%$$

$$\text{DLR}\;(\%)\;=\;\frac{\text{mass}\;\text{of}\;\text{encapsulated}\;\text{melatonin}}{\text{mass}\;\text{of}\;\text{Span}\;60+\text{cholesterol}+\text{melatonin}}\times 100\%$$

where the mass of encapsulated melatonin was calculated from the fluorescence-based standard curve, and the mass of Span 60 + cholesterol + melatonin refers to the total mass of all formulation components used for Nio-MT preparation.

### *In vitro* release of melatonin from Nio-MT

The *in vitro* release behavior of melatonin from Nio-MT was evaluated using a dialysis method. Briefly, 5 mL of the prepared Nio-MT suspension was loaded into a dialysis bag (Solarbio, China, molecular weight cut-off: 8000–14 000 Da), which was then immersed in 2 L of PBS as the release medium. The system was maintained at 37°C under constant stirring at 75 rpm. At predetermined time intervals (every 10 min), 300 μL of the external release medium was collected and equally distributed into three wells (100 μL per well) of a black 96-well plate. An equal volume of fresh PBS (300 μL) was immediately added to maintain a constant total volume. The release process was performed under light-protected conditions by wrapping the containers with aluminum foil.

The fluorescence intensity of each sample was measured using a microplate reader at an excitation wavelength of 285 nm and an emission wavelength of 345 nm. The amount of melatonin released at each time point was calculated according to the previously established standard curve, and the cumulative release profile was then obtained.

### Fabrication of Nio-MT@S-G MN

A 3D-printed PDMS negative mold was used to fabricate a 10 × 10 microneedle array. Briefly, 90 mg GelMA (EFL, China) and 60 mg SilMA (EFL, China) were dissolved in 1 mL of LAP (EFL, China) initiator solution (0.5 mg mL^−^¹ in deionized water) at 45°C until completely dissolved. The resulting solution was then thoroughly mixed with the 100 μL melatonin-loaded niosome suspension to obtain the microneedle precursor solution.

The precursor solution was added dropwise into the PDMS microneedle mold and centrifuged at 2000 × g for 10 min to fill the mold cavities and remove trapped air bubbles. After removal of the excess solution containing bubbles, the remaining solution in the mold was allowed to concentrate at 37°C for 2 h. This loading and centrifugation process was repeated twice to ensure complete formation of the needle tips. Subsequently, a mixed GelMA/SilMA solution without niosomes was added onto the top of the mold to form the backing layer. After centrifugation, the mold was exposed to UV light (405 nm, 8 mW/cm²) for 30 s to induce photocrosslinking. The microneedle patches were then dried at room temperature in the dark for 24 h and carefully peeled from the PDMS mold to obtain the melatonin niosome-loaded microneedle patches.

The morphology of the prepared microneedles was observed using a stereomicroscope and a fluorescence microscope (Zeiss, Germany), and more detailed structural features were further examined by scanning electron microscopy (SEM; Hitachi, Japan).

### Fourier transform infrared spectroscopy

The chemical structures of the individual components and prepared microneedle samples were analyzed by Fourier transform infrared spectroscopy (FTIR). Briefly, the spectra of (GelMA, SilMA, GelMA/SilMA) were recorded using a Nicolet iS20 spectrometer (Thermo Fisher Scientific, USA). The obtained spectra were used to identify characteristic functional groups and to evaluate the interactions among the components in the composite microneedle system.

### *In vitro* dissolution of microneedles

The dissolution behavior of the microneedles was evaluated in PBS under physiological conditions. Briefly, the prepared microneedle patches were immersed in 50 mL PBS and incubated at 37°C without shaking. The samples were collected at 0, 2 and 4 min and observed under a stereomicroscope (Zeiss, Germany). Complete dissolution of the microneedle tips was observed at 4 min.

### Mechanical strength test of microneedles

The mechanical properties of the microneedles were evaluated using an electronic universal testing machine (MTS, USA). Compression was applied using a stainless-steel plate, and force–displacement curves were recorded within a displacement range of 600 μm to assess the mechanical strength of the microneedles.

### Histological evaluation of microneedle penetration

To evaluate the penetration capability of the microneedles, C57BL/6 mice were anesthetized by intraperitoneal injection of Avertin anesthetic (Duowo, China) and then fixed. After the buccal mucosa was exposed with an oral opener and the saliva on the mucosal surface was gently removed with a small cotton ball, the melatonin niosome-loaded microneedle patch was inserted into the buccal mucosa for 1 min. The mice were then euthanized by CO₂ asphyxiation, and the buccal mucosal tissues were collected for HE staining (Solarbio, China).

### Preparation of Nio-MT@S-G MN extracts

To prepare the extracts of Nio-MT@S-G MN, the microneedle patches were first crushed in a 5 mL EP tube. Subsequently, 2 mL of PBS was added, and the mixture was protected from light until the microneedles were completely dissolved. The extract solution was then stored at 4°C for subsequent use.

### Cell culture

HaCaT cells were obtained from Hunan Fenghui Biotechnology Co., Ltd. and cultured according to the manufacturer’s instructions. HaCaT cells were used between passages 3 and 15 for all experiments. Cells were maintained in Dulbecco’s modified Eagle’s medium supplemented with 10% fetal bovine serum (FBS) and 1% penicillin/streptomycin at 37°C in a humidified atmosphere containing 5% CO₂.

### CCK-8 assay

HaCaT cells were seeded into two 96-well plates at a density of 1.0 × 10^5^ cells per well and cultured for 24 h to allow attachment and growth to approximately 80% confluence. The medium was then replaced with fresh medium containing 0, 2, 4, 6, 8 or 10 μL of Nio-MT@S-G MN extract stock solution per well, corresponding to final melatonin-equivalent concentrations of 0, 0.70, 1.37, 2.02, 2.65 and 3.25 μM, respectively. One plate was incubated for 24 h and the other for 48 h. In addition, the cytocompatibility of each individual material component, including Nio, blank SilMA/GelMA microneedle extract (S-G MN), Nio-MT and blank niosome-loaded S-G microneedle extract (Nio@S-G MN), was independently evaluated by CCK-8 assay using their respective concentration gradients (Figure S1, available as supplementary data at *Regenerative Biomaterials* online). After treatment, 10 μL of CCK-8 reagent (MedChemExpress, China) was added to each well and incubated at 37°C for 2 h, followed by measurement of absorbance at 450 nm using a microplate reader. Blank wells containing medium and CCK-8 reagent, but no cells were included for background subtraction prior to analysis.

### Live/dead staining

HaCaT cells were suspended at a density of 4 × 10^5^ cells mL^−1^, and 500 μL of the cell suspension was added to each well of a 24-well plate (*n* = 3). After incubation for 24 h to allow cell attachment, the culture medium was replaced with fresh medium containing the indicated treatments, including PBS, Nio (0.8 μL/100 μL), Nio-MT (71.412 μM), S-G MN (0.577 mg mL^−1^), Nio@S-G MN (8 μL/100 μL) and Nio-MT@S-G MN (1.37 μM). After 24 h of treatment, the cells were rinsed twice with PBS and stained with calcein acetoxymethyl ester (calcein-AM) and PI staining solution (BestBio, China) for 20 min in the dark. Fluorescence images were captured using a laser-scanning confocal microscope (Leica, Germany). For each group, three fields of view were randomly selected and the images were quantitatively analyzed using ImageJ.

### Hemolysis assay

The hemocompatibility of Nio-MT and Nio-MT@S-G MN was evaluated by a hemolysis assay using mouse red blood cells (RBCs). Briefly, anticoagulated whole blood was collected from mice and centrifuged to remove the plasma. The RBCs were resuspended in PBS and washed 3-4 times by repeated centrifugation until the supernatant became clear. The purified RBCs were then resuspended in PBS to obtain a 4% erythrocyte suspension.

For the hemolysis test, 500 μL of the erythrocyte suspension was added into each EP tube. Then, 500 μL ddH₂O was added to the positive control group, 500 μL PBS was added to the negative control group and 500 μL of sample (Nio-MT, Nio-MT @ S-G MN extracts) solution was added to the experimental groups. After incubation at 37°C for 1 h, the samples were centrifuged at 2400 × g for 5 min, and the supernatants were collected. The absorbance of the supernatants was measured at 540 nm using a microplate reader. The hemolysis ratio was calculated according to the following equation:


$$\text{Hemolysis}\;(\%)\;=\;\frac{D540(\text{sample})-D540(\text{negative})}{D540(\text{positive})-D540(\text{negative})}\times 100\%$$

where D540 (sample) is the absorbance of the sample group, D540 (negative) is the absorbance of the negative control group (PBS) and D540 (positive) is the absorbance of the positive control group (ddH₂O).

### Histological analysis

After intraperitoneal injection of PBS, Nio-MT or Nio-MT@S-G MN into 8-week-old female C57BL/6 mice for 14 days, the major organs, including the heart, liver, spleen, lung and kidney, were collected for histological analysis. The tissues were fixed in 4% paraformaldehyde, embedded in paraffin, sectioned into 5 μm slices and stained with HE.

For oral ulcer histological evaluation, buccal mucosal wound tissues were collected from mice at the indicated time point, fixed in 4% paraformaldehyde, embedded in paraffin and sectioned into 5 μm slices. The sections were stained with HE, and histological images were acquired using an Epson V600 digital scanner.

### Bone marrow neutrophil isolation and NETs induction

Mouse bone marrow neutrophils were isolated from femurs and tibias under sterile conditions using density gradient centrifugation. Bone marrow cells were flushed out, passed through a cell strainer and separated with Solutions A/C (Solarbio, China) at 1000 × g for 30 min. The neutrophil fraction was collected, treated with RBC lysis buffer and resuspended in complete medium. To induce NET formation, neutrophils were seeded on poly-L-lysine-coated coverslips at 1 × 10^6^ cells per well and stimulated with ionomycin (2 μM; Sigma-Aldrich, I3909) for 4 h at 37°C under 5% CO₂.

### NETs isolation and quantification

Neutrophils (7 × 10^6^ cells per well) were stimulated with ionomycin (2 μM; Sigma-Aldrich, I3909) at 37°C for 4 h to induce NET formation. The supernatant was carefully discarded, and NETs were collected by washing the culture dishes with pre-cooled PBS. After centrifugation at 450 × g for 5 min at 4°C to remove intact cells, the supernatant was further centrifuged at 18 000 × g for 10 min at 4°C to obtain NETs precipitates. The pellet was resuspended in 100 μL PBS, and NETs concentration was determined by measuring DNA absorbance at 260 nm with a NanoDrop spectrophotometer [49]. The concentration of exogenous NETs (5 μg/mL) was selected based on our preliminary dose-response experiments (1, 2.5, 5 and 10 μg/mL), in which 5 μg/mL achieved optimal inhibition of HaCaT migration without significant cytotoxicity (Figure S2, available as supplementary data at *Regenerative Biomaterials* online).

### Wound healing assay

A scratch wound healing assay was performed to evaluate the effects of the individual components of the Nio-MT@S-G MN system on keratinocyte migration and to determine whether Nio-MT@S-G MN could reverse NETs-mediated inhibition of wound closure. HaCaT cells were seeded into 12-well plates at a density of 5 × 10^5^ cells per well and cultured to confluence. A uniform linear scratch was created in each well using a sterile pipette tip, and detached cells were removed by gentle washing with PBS.

For component comparison, cells were treated with PBS, Nio (0.8 μL/100 μL), Nio-MT (714.12 μM), S-G MN (0.577 mg mL^−1^), Nio@S-G MN (8 μL/100 μL) and Nio-MT@S-G MN (13.7 μM/mL). To assess the effect of NETs on keratinocyte migration and the potential therapeutic rescue by the microneedle formulation, HaCaT cells were first exposed to cell-free NETs prepared from ionomycin-stimulated neutrophils, followed by treatment with Nio-MT@S-G MN. Images of the wounded areas were captured at 0, 12 and 24 h at pre-marked positions under a microscope. The wound area at each time point was quantified using ImageJ software, and wound closure was expressed as the relative decrease in wound area compared with that at 0 h.

### Transwell migration assay

HaCaT cells were suspended in serum-free medium and seeded into the upper chambers of 8 μm PET Transwell inserts at a density of 5 × 10^4^ cells per insert. The lower chambers were filled with complete medium containing 10% FBS as a chemoattractant.

For the NETs group, cell-free NETs were added to the upper chamber at a final concentration of 5 μg mL^−^¹. For the treatment group, HaCaT cells were co-incubated with NETs and Nio-MT@S-G MN extracts (13.7 μM) in the upper chamber. The control group received serum-free medium without NETs or extracts. After incubation for 48 or 72 h at 37°C in 5% CO₂, the non-migrated cells on the upper surface of the membrane were gently removed with cotton swabs. The migrated cells on the lower surface were fixed with 4% paraformaldehyde for 10 min, stained with 0.1% crystal violet (Solarbio, China) for 15 min and then imaged under a microscope. Cell migration was quantified by counting the migrated cells in three random fields per insert using ImageJ software.

### Immunofluorescence staining of ZO-1

Sterile glass coverslips were placed in 24-well plates after ethanol and UV sterilization. HaCaT cells were seeded onto the coverslips at a density of 5 × 10^4^ cells per well and cultured for 24 h. The cells were then assigned to three groups: CTR, NETs and NETs + Nio-MT@S-G MN. Cells in the CTR group were treated with PBS, whereas cells in the NETs group were treated with 5 μg mL^−^¹ cell-free NETs. For the NETs + Nio-MT@S-G MN group, cells were co-treated with 5 μg mL^−^¹ NETs and 13.7 μM Nio-MT@S-G MN extracts. After 24 h of treatment, the cells were washed with PBS and fixed with 4% paraformaldehyde for 10 min. The cells were then permeabilized with 0.3% Triton X-100 for 15 min and blocked with 5% donkey serum for 1 h at room temperature.

The cells were subsequently incubated with a rabbit anti-ZO-1 primary antibody (Thermo Fisher, Cat. No. 40-2200; 1:600) overnight at 4°C. After washing with PBS, the cells were incubated with an Alexa Fluor 555-conjugated donkey anti-rabbit secondary antibody (Beyotime, Cat. No. A0453; 1:300) for 1 h at room temperature in the dark. The nuclei were counterstained with DAPI (Beyotime, Cat. No.C1002; 1:1000) for 5 min. After PBS washing, the coverslips were mounted using an anti-fade mounting medium. Fluorescence images were acquired under identical settings for all groups.

### Immunofluorescence staining for NETs

Neutrophils stimulated with ionomycin (4 h) were fixed with 4% PFA (10 min), permeabilized with 0.2% Triton X-100 (15 min) and blocked with 5% donkey serum (1 h). Primary antibodies (CitH3 1:600, Abcam, ab5103) were incubated overnight at 4°C, followed by species-matched Alexa Fluor secondary antibodies (1:300, 1.5 h). Nuclei were counterstained with DAPI (1:1000, 5 min, Beyotime), with PBST washes between steps.

### SYTOX green staining

SYTOX Green staining was carried out in the dark. For microscopy, cells were stained with SYTOX Green (Thermo Fisher Scientific, S7020; 1:50 000) for 10 min at room temperature, washed three times with PBS and imaged using FITC/GFP filters (Ex/Em = 504/523 nm). For flow cytometry, ionomycin-stimulated neutrophils (4 μM) were stained with SYTOX Green at 1 drop per 1 mL of cell suspension (1 × 10^7^ cells mL^−^¹) for 10 min in the dark and analyzed directly without washing.

### Reverse transcription‑quantitative real‑time PCR

Total RNA was extracted from cells and tissue samples using TRIzol reagent (Takara, Japan) according to the manufacturer’s protocol. Briefly, samples were lysed in TRIzol, mixed with chloroform and centrifuged at 12 000 × g for 15 min at 4°C to separate the aqueous phase. The RNA was then precipitated, washed with ethanol and dissolved in RNase-free water (Takara, Japan). RNA concentration and purity were determined using a NanoDrop spectrophotometer (Thermo, USA).

For reverse transcription, 500 ng of total RNA was converted into complementary DNA using a reverse transcription kit (Takara, Japan) on a thermal cycler (ProFlex, Thermo, USA). Quantitative real-time PCR was performed using Power SYBR™ Green PCR Master Mix (Thermo Fisher Scientific, USA) on a 7500 Real-Time PCR System (Applied Biosystems, USA). The primer sequences for interleukin-1β (IL-1β), tumor necrosis factor-α (TNF-α), PAD4, neutrophil elastase (Elane) and ZO-1 are listed in Table S3, available as supplementary data at *Regenerative Biomaterials* online. β-actin was used as the internal reference, and relative gene expression was calculated using the 2^−ΔΔCt^ method. Melt curve analysis was performed to confirm amplification specificity. All samples were analyzed in triplicate.

### Western blot

Total protein was extracted from cells and tissue samples using RIPA buffer containing protease inhibitors (Beyotime, P0013B). Protein concentration was measured with a BCA assay kit (Beyotime, P0011). Equal amounts of protein were resolved by SDS-PAGE on self-cast 12.5% or 7.5% gels and transferred to membranes. After blocking, the membranes were incubated overnight at 4°C with primary antibodies against CitH3 (Abcam, ab5103, 1:1000) and PAD4 (Abcam, ab214810, 1:1000), followed by incubation with HRP-conjugated secondary antibodies for 1 h at room temperature. Bands were detected using a chemiluminescent substrate (Merck Millipore) and imaged on a ChemiDoc MP Imaging System (Bio-Rad).

### Statistical analysis

All data are presented as mean ± standard error of the mean (SE). Statistical analyses were performed using Prism 9 (GraphPad Software, USA). Flow cytometry data were processed and visualized with FlowJo software. Normality of data distribution was assessed using the Shapiro-Wilk test prior to statistical analysis. Two-group comparisons were analyzed by two-tailed unpaired Student’s *t*-tests. For comparisons among multiple groups, one-way ANOVA or the nonparametric Kruskal–Wallis test was applied, followed by Tukey’s or Bonferroni’s *post hoc* tests as appropriate. A significance threshold of *P* < 0.05 was used for all analyses. Statistical significance is indicated in the figures as follows: *P < 0.05, ^#^*P* < 0.05, **P < 0.01, ^##^*P* < 0.01, ***P < 0.001; ^###^*P* < 0.001, ****P < 0.0001 and “ns” denotes not significant.

## Results

### Identification of neutrophil-associated pathology in sleep deprivation-aggravated oral ulcers and construction/characterization of Nio-MT@S-G MN

To preliminarily determine whether neutrophil-derived inflammatory activity is enhanced in sleep deprivation-aggravated oral ulcers, we first compared OU group with OUS group. Immunofluorescence staining of ulcer tissues on Day 6 showed markedly stronger CitH3/Myeloperoxidase (MPO) co-localization in the OUS group than in the OU group, indicating increased NETs-associated inflammatory accumulation under sleep-deprived conditions (Figure 1A). Consistently, quantitative real‑time PCR (qPCR) analysis further revealed significant upregulation of the NET-related markers PAD4 and Elane in the OUS group (Figure 1B). These findings suggest that neutrophil-associated pathology is aggravated in sleep deprivation-associated oral ulcers and support neutrophil-derived NETs as a relevant therapeutic target.

**Figure 1:**
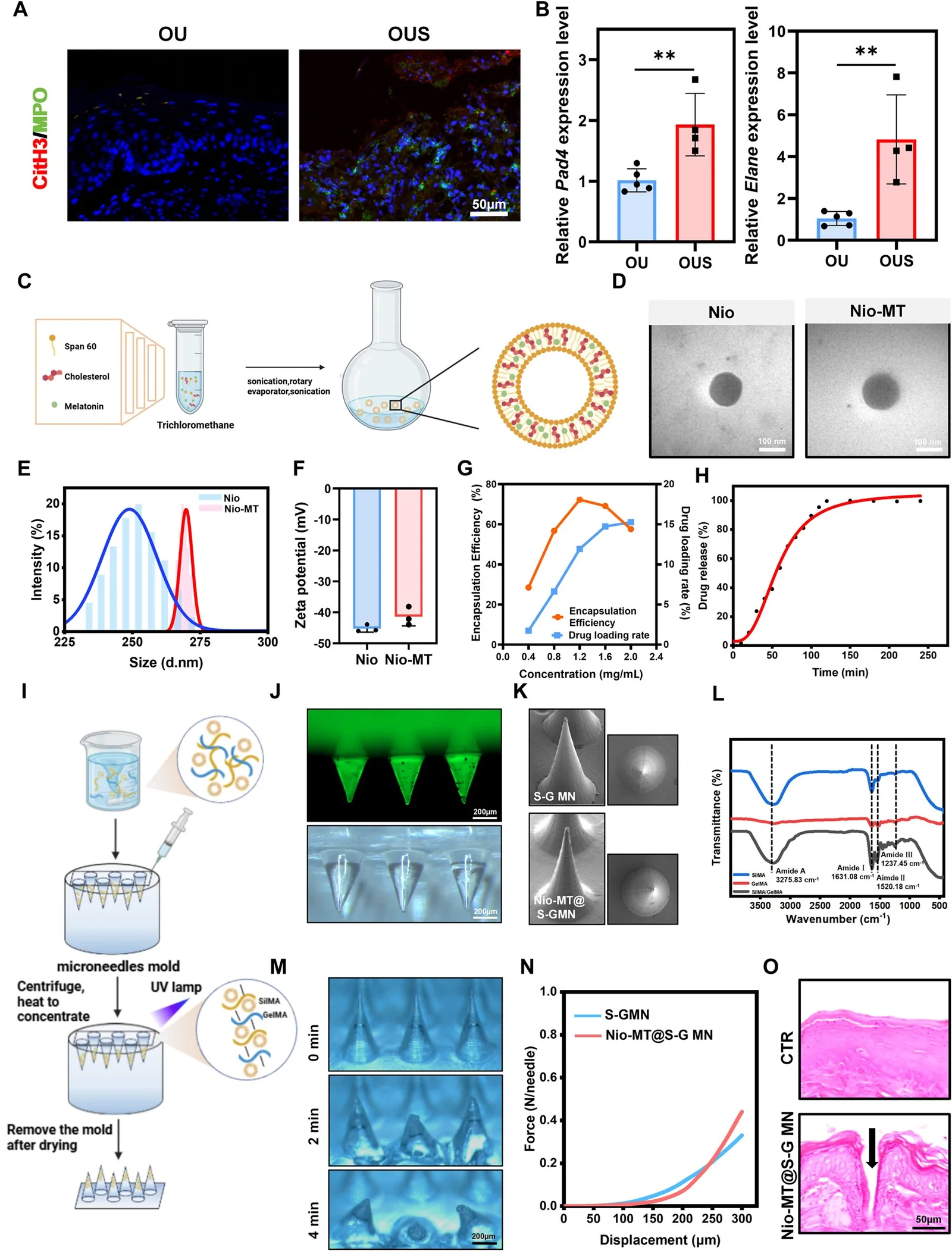
Identification of neutrophil-associated pathology in sleep deprivation-aggravated oral ulcers and the construction/characterization of Nio-MT@S-G MN. **(A)** Immunofluorescence staining of CitH3 (red) and MPO (green) in oral ulcer tissues from the OU and OUS groups on Day 6. Nuclei were counterstained with DAPI (blue). Scale bar: 50 μm. **(B)** Relative mRNA expression levels of the NET-related markers PAD4 and Elane in ulcer tissues from the OU and OUS groups. **(C)** Schematic illustration of the preparation process of melatonin-loaded niosomes (Nio-MT). **(D)** TEM images of blank niosomes and Nio-MT. Scale bar: 100 nm. **(E)** Particle size distribution of blank niosomes and Nio-MT. **(F)** Zeta potential of blank niosomes and Nio-MT. **(G)** Encapsulation efficiency and drug loading of Nio-MT at different melatonin concentrations. **(H)** *In vitro* cumulative release profile of melatonin from Nio-MT. **(I)** Schematic illustration of the fabrication process of Nio-MT@S-G MN**. (J)** Representative fluorescence and bright-field images of the microneedle array. Scale bar: 200 μm. **(K)** SEM images of S-G MN and Nio-MT@S-G MN. **(L)** FTIR spectra of SilMA, GelMA, and the composite microneedle system. **(M)** *In vitro* dissolution behavior of the microneedles in PBS at 0, 2 and 4 min. Scale bar: 0.2 mm. **(N)** Mechanical strength test of the microneedles shown as force–displacement curves. **(O)** HE staining of mouse buccal mucosa after microneedle application, showing successful mucosal penetration. Scale bar: 50 μm. Data are presented as mean ± SD. Statistical significance was analyzed as indicated in the Section Methods. **P < 0.01.

Based on this rationale, melatonin-loaded niosomes (Nio-MT) were prepared by the thin-film hydration method (Figure 1C) [50, 51]. TEM analysis showed that both empty and melatonin-loaded niosomes exhibited a regular spherical morphology with uniform dispersion (Figure 1D). Upon melatonin encapsulation, the average particle size increased slightly from approximately 250 nm to about 275 nm, while remaining within the nanoscale range, as determined by dynamic light scattering (Figure 1E). Zeta potential analysis revealed a stable surface charge, indicating satisfactory colloidal stability of the niosomes (Figure 1F). Encapsulation studies further showed that Nio-MT achieved optimal encapsulation efficiency and relatively high drug loading at a melatonin concentration of 1.2 mg/mL (Figure 1G). *In vitro* release assays demonstrated a sustained and stable release profile, with melatonin release gradually reaching a plateau at approximately 180 min (Figure 1H).

Subsequently, Nio-MT were embedded into a SilMA/GelMA hydrogel matrix to construct the Nio-MT@S-G MN microneedle system (Figure 1I) [52, 53]. The microneedle arrays possessed a well-defined, regularly arranged geometry under fluorescence microscopy and stereomicroscopy, and SEM further confirmed that the microneedles had intact, sharp tips with a length of 600 μm and well-formed structures (Figure 1J and K). FTIR analysis of the SilMA/GelMA composite hydrogel revealed characteristic absorption bands at 1631.08 cm^−^¹ (amide I, C = O stretching), 1520.18 cm^−^¹ (amide II, N-H bending), 1237.45 cm^−^¹ (amide III, C-N stretching) and 3275.83 cm^−^¹ (amide A, N-H stretching). The amide I band at 1631.08 cm^−^¹ falls within the characteristic range for β-sheet conformation of silk fibroin (1620–1640 cm^−^¹), while the amide A band confirms the presence of hydrogen bonding interactions [54]. These spectral features indicate the successful incorporation of silk fibroin-based components and the preservation of β-sheet secondary structures (Figure 1L). In a PBS degradation assay, the microneedle tips rapidly disintegrated upon contact with PBS, with nearly complete dissolution observed within 4 min (Figure 1M), supporting their rapid responsiveness for local drug release after insertion. Mechanical compression tests showed that both S-G MN and Nio-MT@S-G MN displayed progressively increased loading force with increasing displacement. An obvious force instability was observed in the S-G MN group at approximately 300 μm, whereas Nio-MT@S-G MN maintained a continuous increase in force and exhibited a higher loading force at larger displacements (Figure 1N). Consistently, *in vivo* application on mouse oral mucosa demonstrated effective disruption of the keratinized layer and formation of distinct microchannels, as confirmed by HE staining (Figure 1O), verifying the favorable tissue penetration capability of the Nio-MT@S-G MN system.

### Biocompatibility and biosafety of Nio-MT@S-G MN

To investigate the good biocompatibility of Nio-MT@S-G MN, we tried to validate them *in vitro* and *in vivo* [55]. Wound healing is a continuous and dynamic process consisting of hemostasis, inflammation, proliferation and remodeling, in which keratinocytes play a critical role in re-epithelialization and cell migration [56, 57]. Therefore, HaCaT cells were first incubated with extracts of Nio-MT@S-G MN at different concentrations for 24 and 48 h. CCK-8 assays showed that cell viability remained above 90% under all tested conditions (Figure 2A), indicating negligible cytotoxicity of the microneedle system. Consistently, live/dead staining further confirmed the favorable cytocompatibility. A large proportion of viable cells (green fluorescence) was observed in all groups, with cell survival rates exceeding 80% (Figure 2B). Quantitative analysis demonstrated no significant differences in cell viability among the different treatment groups (Figure 2C), suggesting that the Nio-MT@S-G MN extracts did not adversely affect keratinocyte viability.

**Figure 2:**
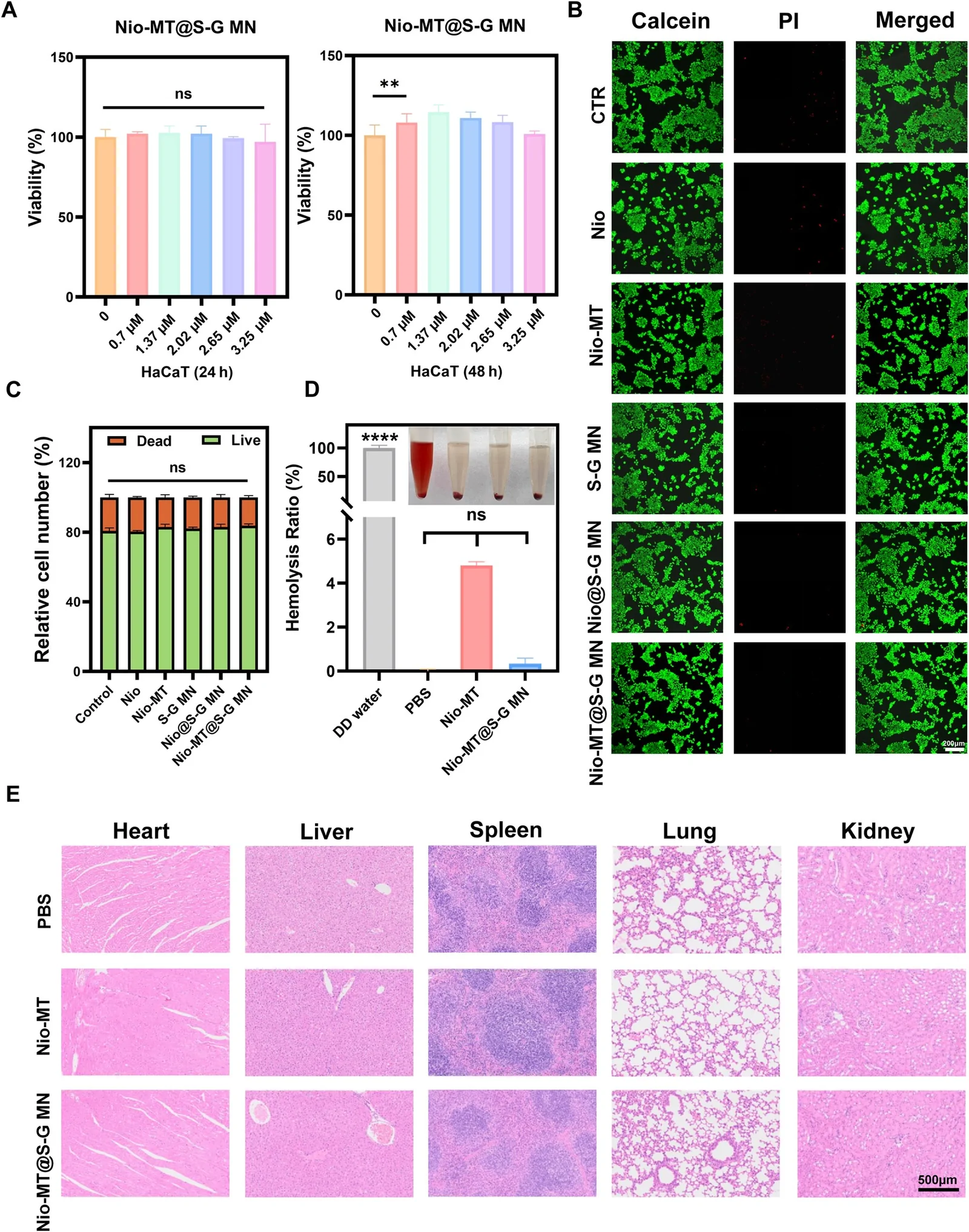
Biocompatibility and biosafety of Nio-MT@S-G MN. **(A)** CCK-8 assay of HaCaT cells treated with different concentrations of Nio-MT@S-G MN for 24 and 48 h, showing cell viability under each condition. **(B)** Representative live/dead staining images of HaCaT cells treated with CTR, Nio, Nio-MT, S-G MN, Nio@S-G MN and Nio-MT@S-G MN. Live cells are stained green with calcein-AM and dead cells are stained red with PI. Scale bar: 400 μm. **(C)** Quantitative analysis of the relative percentages of live and dead cells in the different treatment groups shown in B. **(D)** Hemolysis assay of ddH₂O (positive control), PBS (negative control), Nio-MT and Nio-MT@S-G MN. Insets show representative photographs of RBC suspensions after treatment. **(E)** HE staining of major organs, including the heart, liver, spleen, lung and kidney, collected from mice treated with PBS, Nio-MT or Nio-MT@S-G MN for systemic biosafety evaluation. Scale bar: 500 μm. Data are presented as mean ± SD. Statistical significance was analyzed as described in the Section Methods. **P < 0.01; ****P < 0.0001; ns, not significant.

Hemocompatibility was subsequently evaluated using a hemolysis assay. The hemolysis rates of Nio-MT and Nio-MT@MN were comparable to those of the negative control (PBS) and were markedly lower than those of the positive control (ddH_2_O; Figure 2D), indicating excellent hemocompatibility of the system. Furthermore, *in vivo* toxicity was assessed by intraperitoneally injecting mice with Nio-MT, S-G MN or Nio-MT@S-G MN. After 2 weeks, HE staining revealed intact histological structures without obvious inflammatory infiltration or necrosis in major organs (Figure 2E), demonstrating the favorable biosafety of each component in the Nio-MT@S-G MN system. Collectively, these findings demonstrate that Nio-MT@S-G MN exhibits excellent biocompatibility and biosafety both *in vitro* and *in vivo*.

### *In vitro* effects of Nio-MT@S-G MN on NET formation

Given that sleep deprivation primes neutrophils for heightened NET formation, we first examined whether Nio‑MT@S‑G MN could modulate the NET‑related molecular program. qPCR showed that PAD4 and Elane were elevated in sleep deprivation (SD) neutrophils at baseline and were further induced after stimulation, whereas Nio-MT@S-G MN treatment attenuated these increases, with a stronger effect in SD than in CTR (Figure 3A and B). Western blot analysis confirmed that PAD4 and CitH3 were upregulated in SD neutrophils and were reduced by Nio-MT@S-G MN (Figure 3C). Functionally, immunofluorescence indicated diminished CitH3 deposition after treatment, and SYTOX Green staining together with flow cytometry showed reduced extracellular DNA release (Figure 3D–H). To rule out cytotoxicity as the cause of reduced SYTOX Green signal, CCK-8 assays confirmed that Nio-MT@S-G MN did not affect neutrophil viability at the tested concentrations (Figure S3, available as supplementary data at *Regenerative Biomaterials* online). These results suggest that Nio-MT@S-G MN effectively suppresses excessive NET formation, particularly under sleep deprivation-associated conditions.

**Figure 3:**
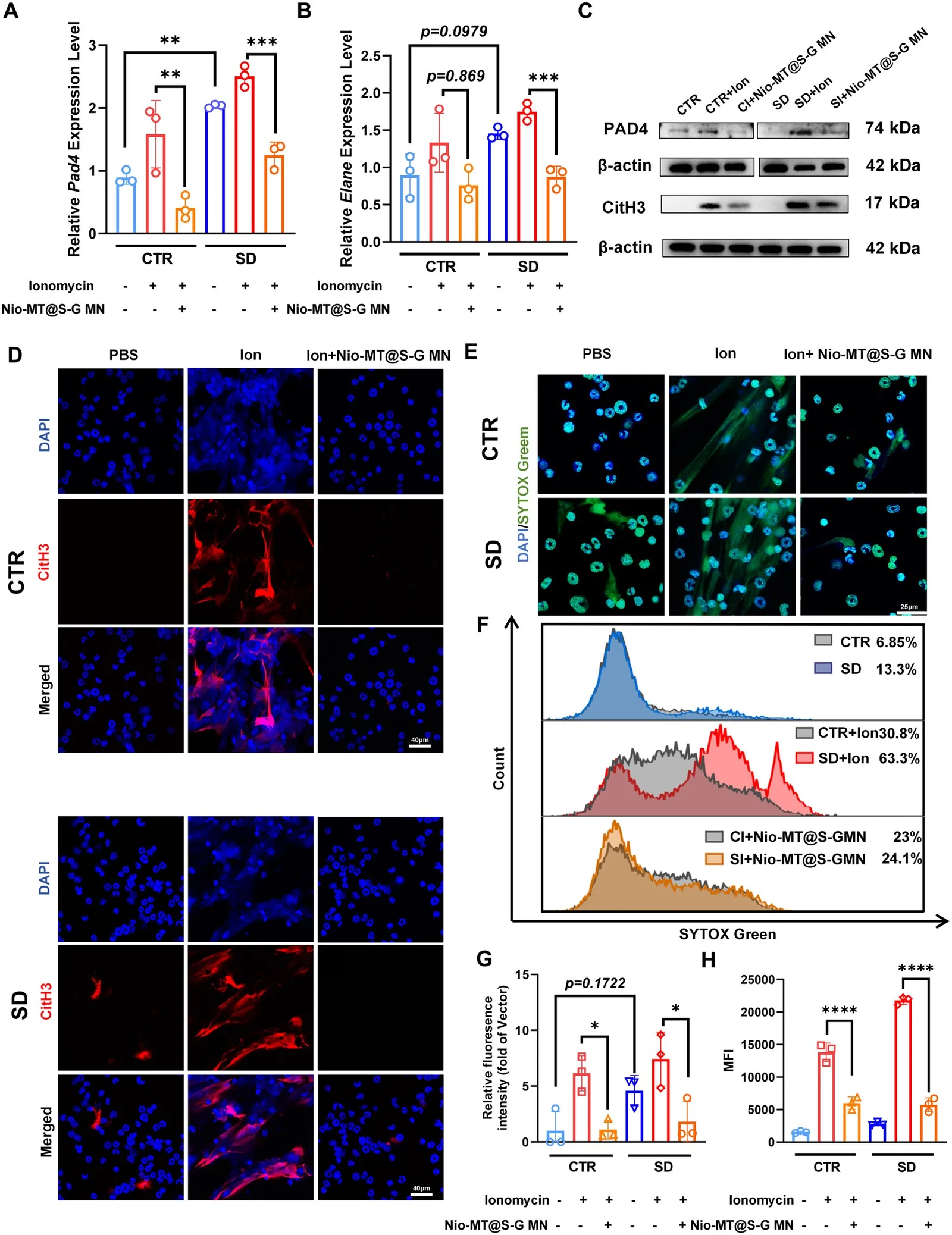
*In vitro* effects of Nio-MT@S-G MN on NET formation. **(A, B)** Relative mRNA expression levels of the NET-related markers PAD4 **(A)** and Elane **(B)** in neutrophils isolated from CTR and SD mice under the indicated treatments. Neutrophils were stimulated with ionomycin with or without Nio-MT@MN treatment. **(C)** Western blot analysis of PAD4 and CitH3 protein expression in neutrophils from the CTR and SD groups under the indicated treatments. β-actin was used as the loading control. **(D)** Immunofluorescence staining of CitH3 (red) in neutrophils from the CTR and SD groups treated with PBS, ionomycin (ion) or ionomycin + Nio-MT@S-G MN. Nuclei were counterstained with DAPI (blue). Scale bar: 40 μm. **(E)** Representative fluorescence images of extracellular DNA release detected by SYTOX green staining in neutrophils from the CTR and SD groups treated with PBS, ionomycin (ion) or ionomycin + Nio-MT@S-G MN. Nuclei were counterstained with DAPI. Scale bar: 25 μm. **(F)** Representative flow cytometry histograms of SYTOX green fluorescence in neutrophils from the CTR and SD groups under the indicated treatments. **(G)** Quantification of the relative fluorescence intensity of CitH3 staining shown in D. **(H)** Quantification of the mean fluorescence intensity (MFI) of SYTOX green shown in F, reflecting extracellular DNA release/NET formation. Data are presented as mean ± SD. Statistical significance was analyzed as described in the Section Methods. *P < 0.05, **P < 0.01, ***P < 0.001, ****P < 0.0001.

### Nio-MT@S-G MN promotes keratinocyte migration and counteracts NETs-mediated inhibition

Since NETs accumulation impairs epithelial migration [15], we evaluated the capacity of Nio‑MT@S‑G MN to rescue cellular functionality. Under basal conditions, scratch assays revealed that Nio‑MT@S‑G MN promoted HaCaT cell migration, showing the highest wound closure rate at 24 h among all groups (Figure 4A and B). To establish a model of NETs‑induced impairment on cell migration, HaCaT cells were exposed to ionomycin‑induced NETs. NETs exposure markedly impaired wound closure at 12 and 24 h. However, treatment with Nio‑MT@S‑G MN extracts substantially restored migratory capacity (Figure 4C–E). Transwell assays further confirmed that Nio‑MT@S‑G MN reversed the inhibition of transmembrane migration induced by NETs (Figure 4F and G).

**Figure 4:**
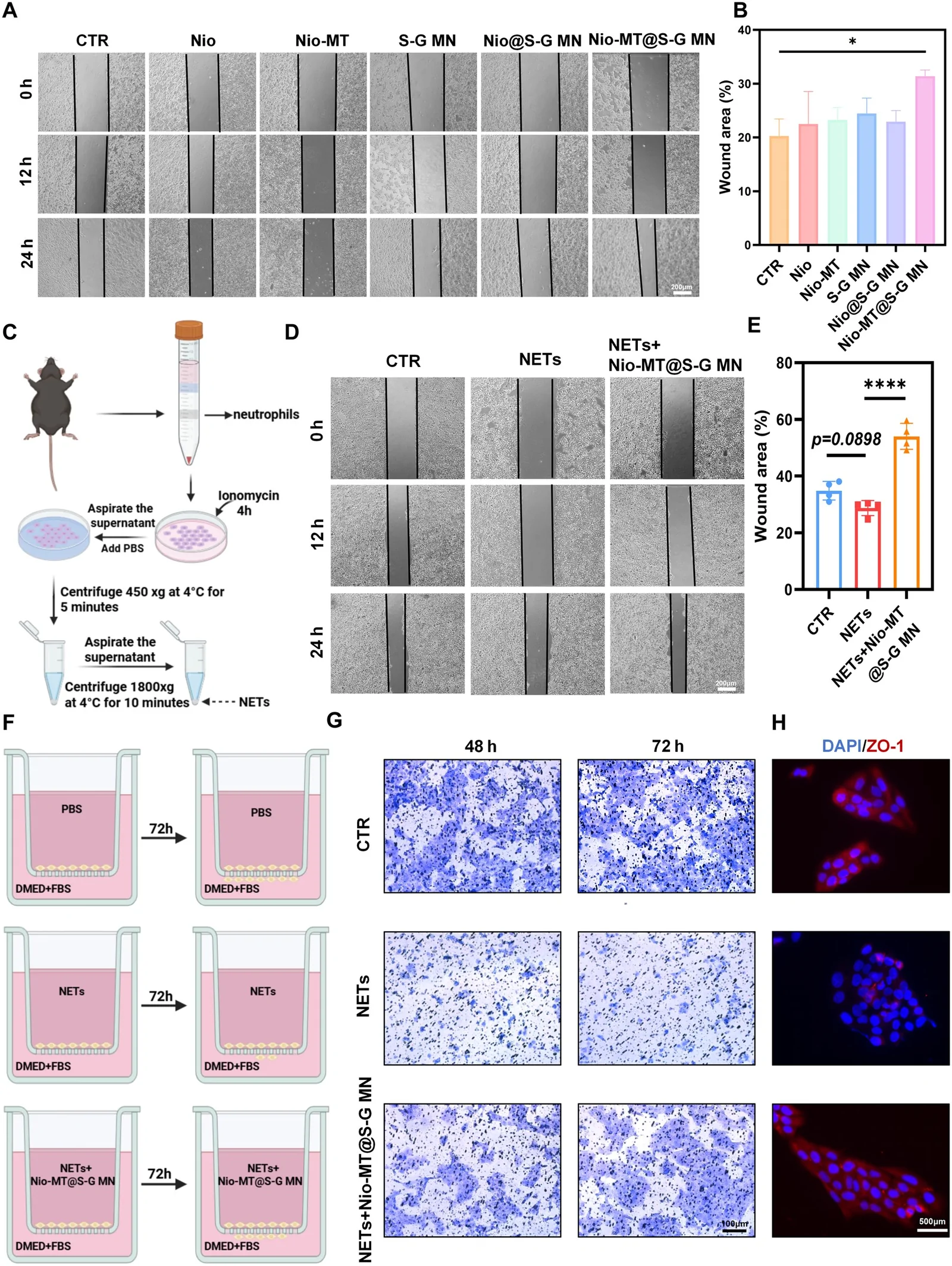
Nio-MT@S-G MN Promotes keratinocyte migration and counteracts NETs-mediated inhibition. **(A)** Representative scratch wound images of HaCaT cells treated with CTR, Nio, Nio-MT, S-G MN, Nio@S-G MN or Nio-MT@S-G MN at 0, 12 and 24 h. Scale bar: 200 μm. **(B)** Quantification of wound area in the different treatment groups shown in A. **(C)** Schematic illustration of bone marrow neutrophil isolation and preparation of cell-free NETs from ionomycin-stimulated neutrophils. **(D)** Representative scratch wound images of HaCaT cells treated with CTR, NETs or NETs + Nio-MT@S-G MN at 0, 12 and 24 h. Scale bar: 200 μm. **(E)** Quantification of wound area in the groups shown in D. **(F)** Schematic illustration of the transwell migration assay used to evaluate the effect of NETs and Nio-MT@S-G MN on HaCaT cell migration. **(G)** Representative crystal violet staining images of migrated HaCaT cells in the CTR, NETs and NETs + Nio-MT@S-G MN groups at 48  and 72 h. Scale bar: 100 μm. **(H)** Immunofluorescence staining of ZO-1 (red) in HaCaT cells treated with CTR, NETs or NETs + Nio-MT@S-G MN. Nuclei were counterstained with DAPI (blue). scale bar: 500 μm. Data are presented as mean ± SD. Statistical significance was analyzed as described in the Section Methods. *P < 0.05, ****P < 0.0001.

We then investigated whether this protective effect extended to epithelial barrier function. Immunofluorescence staining for ZO-1 showed that NETs exposure markedly reduced ZO-1 fluorescence intensity and disrupted intercellular junction continuity (Figure 4H). In contrast, treatment with Nio-MT@S-G MN restored ZO-1 distribution and enhanced fluorescence intensity. Western blot analysis further confirmed that NETs exposure reduced ZO-1 protein expression, which was restored upon Nio-MT@S-G MN treatment (Figure S4, available as supplementary data at *Regenerative Biomaterials* online). Annexin V-FITC/PI flow cytometry confirmed that NETs did not induce apoptosis in HaCaT cells (Figure S5, available as supplementary data at *Regenerative Biomaterials* online), indicating that the impaired migration is due to functional inhibition rather than cell death. Collectively, these results demonstrate that Nio-MT@S-G MN effectively preserves epithelial barrier integrity and mitigates NETs-mediated structural damage.

### *In vivo* evaluation of Nio-MT@S-G MN in alleviating sleep deprivation -aggravated oral ulcers

Guided by the *in vitro* findings that sleep deprivation enhanced NET formation and that Nio-MT@S-G MN effectively suppressed ionomycin-induced NETs overproduction, its therapeutic performance was next evaluated in a chemical-induced oral ulcer model under sleep deprivation (Figure 5A). Mice were divided into three groups: OU, OUS and OUS+Nio-MT@S-G MN.

**Figure 5:**
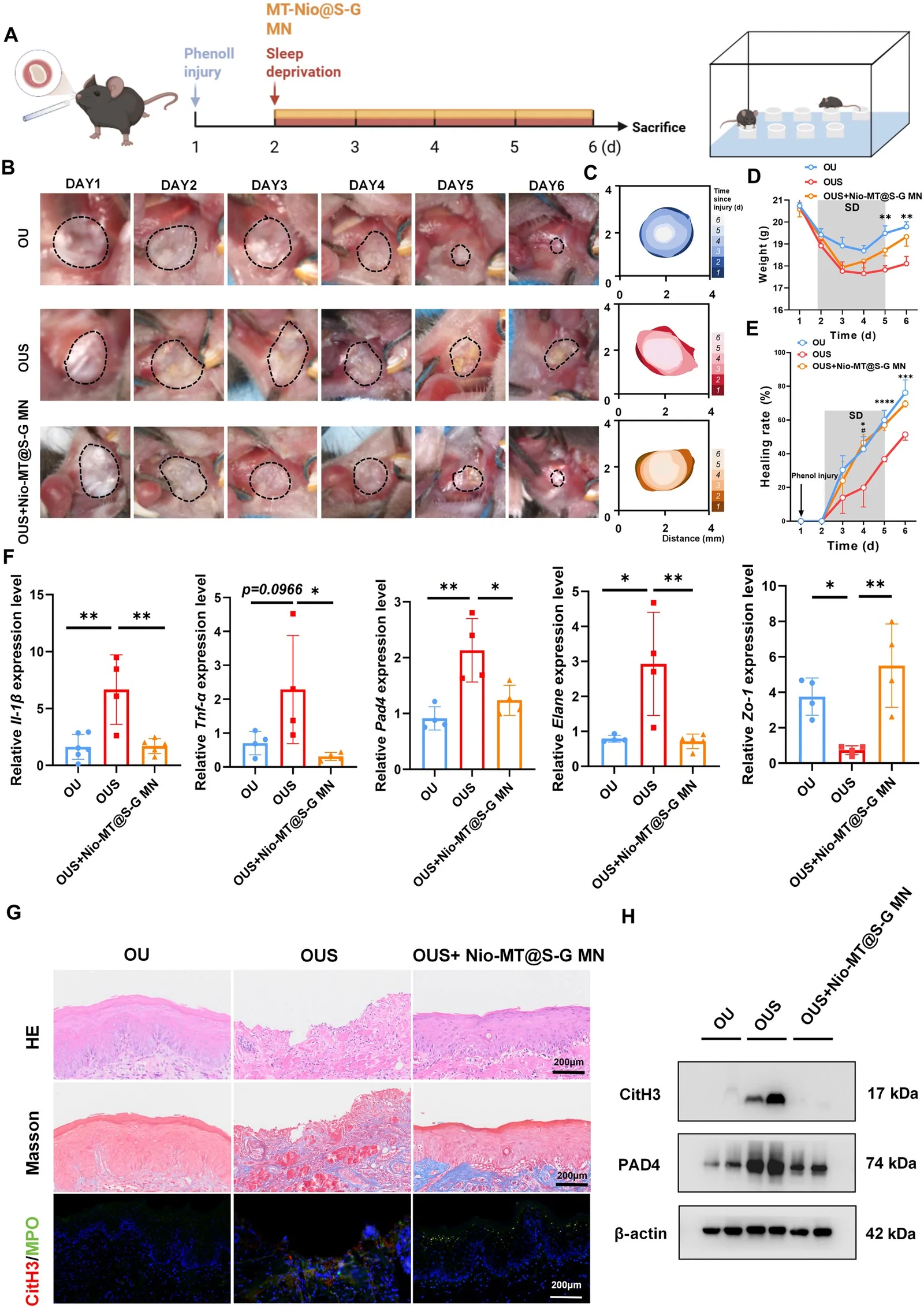
*In vivo* evaluation of Nio-MT@S-G MN in alleviating sleep deprivation-aggravated oral ulcers. **(A)** Schematic illustration of the experimental design for the chemical-induced oral ulcer model under sleep deprivation and Nio-MT@S-G MN treatment. **(B)** Representative macroscopic images of oral ulcers in the OU, OUS and OUS+Nio-MT@S-G MN groups from Day 1 to Day 6. Dashed lines indicate the wound margins. **(C)** Representative wound closure trajectories of individual mice in the three groups over time. **(D)** Body weight changes of mice in the OU, OUS and OUS+Nio-MT@S-G MN groups during the experimental period. **(E)** Quantification of oral ulcer healing rate in the three groups over time. **(F)** Relative mRNA expression levels of IL-1β, TNF-α, PAD4, Elane and ZO-1 in oral ulcer tissues from the indicated groups. **(G)** Histological, histochemical and immunofluorescence analyses of oral ulcer tissues. Upper panel: representative HE staining images showing epithelial morphology and inflammatory infiltration. Middle panel: Masson’s trichrome staining for collagen deposition (blue) in the healed mucosa at Day 6. Lower panel: immunofluorescence staining of CitH3 (red) and MPO (green), with nuclei counterstained with DAPI (blue), showing NET-associated deposition in ulcer tissues. Scale bar: 200 μm. **(H)** Western blot analysis of CitH3 and PAD4 protein expression in oral ulcer tissues from the indicated groups. β-actin was used as the loading control. Data are presented as mean ± SD. **(D)** and **(E)** * indicates the difference between the OU and OUS groups, and # indicates the difference between the OU and OUS+Nio-MT@S-G MN groups. Statistical significance was analyzed as described in the Section Methods. *P < 0.05, ^#^*P* < 0.05, **P < 0.01, ***P < 0.001, ****P < 0.0001.

Gross observation of the buccal mucosa showed that ulcer closure was markedly delayed by sleep deprivation (Figure 5B). Compared with the OU group, larger residual lesions persisted in the OUS group throughout the observation period. In contrast, treatment with Nio-MT@S-G MN visibly accelerated wound closure under sleep-deprived conditions. This trend was further supported by representative wound-closure trajectories from individual mice, in which lesions in the OU group were nearly resolved by Day 6, whereas obvious unhealed areas remained in the OUS group; notably, the OUS+Nio-MT@S-G MN group displayed a substantially smaller residual wound area and a healing pattern closer to that of the OU group (Figure 5C).

A progressive loss of body weight was also induced by sleep deprivation, indicating that ulcer healing under this condition was accompanied by impaired systemic physiological status (Figure 5D). This decline was partially alleviated after Nio-MT@S-G MN treatment. Consistently, quantitative analysis demonstrated that the wound healing rate was significantly reduced in the OUS group relative to the OU group, whereas administration of Nio-MT@S-G MN markedly improved ulcer closure, particularly during the later phase of repair (Figure 5E).

To determine whether these therapeutic effects were associated with modulation of local inflammation and NET-related pathology, ulcer tissues were further analyzed at the molecular and histological levels. qPCR analysis revealed that sleep deprivation significantly upregulated the proinflammatory cytokines IL-1β and TNF-α, as well as the NET-associated markers PAD4 and Elane, while suppressing expression of the tight junction protein ZO-1 (Figure 5F). These alterations were substantially reversed by Nio-MT@S-G MN treatment, suggesting simultaneous attenuation of inflammatory activation, restriction of NET-associated dysregulation and recovery of barrier-related gene expression.

Histological, histochemical and immunofluorescence analyses yielded consistent results (Figure 5G). Severe epithelial disruption together with pronounced inflammatory cell infiltration was observed in the OUS group by HE staining, whereas improved mucosal continuity and reduced inflammatory infiltration were evident after Nio-MT@S-G MN treatment. Masson’s trichrome staining further revealed that collagen deposition in the healed mucosa was markedly reduced and disorganized in the OUS group, while Nio-MT@S-G MN treatment significantly restored collagen content and fibrillar organization, indicating improved tissue remodeling quality. Meanwhile, strong co-localization of CitH3 and MPO was detected in ulcer tissue from the OUS group, indicating abundant NETs deposition *in vivo*. This signal was markedly diminished in the treated group, supporting effective suppression of aberrant NET formation by Nio-MT@S-G MN. Western blot analysis further confirmed these findings, as elevated CitH3 and PAD4 protein levels in the OUS group were both markedly reduced following treatment (Figure 5H). To verify the NETs-dependent mechanism, we included the PAD4 inhibitor Cl-amidine as a positive control (Figure S5, available as supplementary data at *Regenerative Biomaterials* online), which reduced CitH3/PAD4 expression and accelerated wound closure, recapitulating the effects of Nio-MT@S-G MN. Furthermore, control experiments with individual components (free melatonin, blank niosomes and blank S-G microneedles) confirmed that the full integrated system is required for therapeutic efficacy (Figure S6, available as supplementary data at *Regenerative Biomaterials* online).

Taken together, these results demonstrate that Nio-MT@S-G MN effectively rescues sleep deprivation-impaired oral ulcer healing by accelerating wound closure, reducing inflammatory burden, suppressing NETs deposition and promoting restoration of mucosal barrier integrity.

## Discussion

Sleep disturbances are increasingly prevalent in modern society, and their impact on immune homeostasis and tissue repair has attracted growing attention [58]. Although sleep deprivation has been reported to aggravate oral ulcers and delay healing, the local pathological features that distinguish sleep deprivation-associated oral ulcers from conventional lesions have remained insufficiently defined [4]. In the present study, both tissue-level and molecular analyses support the view that sleep deprivation reshapes the inflammatory microenvironment of oral ulcers. Specifically, immunofluorescence staining on Day 6 revealed markedly increased CitH3/MPO co-localization in the OUS group relative to the OU group, while qPCR analysis showed significant upregulation of the NET-associated markers PAD4 and Elane. These results indicate that sleep deprivation is accompanied by enhanced neutrophil-associated inflammatory activity in oral ulcer tissues, suggesting that NET-related pathology may be an important feature of delayed healing under sleep-disturbed conditions. This observation suggests that oral ulcers in the context of sleep disturbance are not merely slower healing lesions, but rather a distinct pathological setting accompanied by amplified neutrophil-derived inflammatory responses. On this basis, targeting NET-associated pathology appears to be a rational therapeutic strategy for this underrecognized condition. Melatonin was selected as the active agent for this purpose based on its well-established capacity to counteract NET formation. Mechanistically, melatonin suppresses NETs through convergent pathways: it scavenges ROS, intercepting the upstream oxidative trigger of NETosis [59], and inhibits the NF-κB signaling pathway, reducing the pro-inflammatory transcriptional program that primes neutrophils for NET formation [60]. These mechanisms converge to suppress the PAD4/CitH3 axis and reduce extracellular DNA release, consistent with the reductions observed in our study. Accordingly, we developed a melatonin-loaded niosome–hydrogel microneedle system (Nio-MT@S-G MN) to locally modulate the pathological microenvironment of sleep deprivation-aggravated oral ulcers.

Our *in vitro* data further support the relevance of this strategy. Exogenous NETs markedly impaired keratinocyte migration and reduced the expression of the tight junction protein ZO-1, whereas treatment with Nio-MT@S-G MN alleviated these effects. These findings indicate that NET-associated inflammation not only amplifies tissue injury but may also directly compromise epithelial barrier restoration by disrupting keratinocyte motility and cell–cell junction integrity. In parallel, Nio-MT@S-G MN reduced extracellular DNA release and downregulated NETs-associated markers, including CitH3 and PAD4, in ionomycin-stimulated neutrophils, supporting its ability to suppress excessive NET formation. Although the precise molecular pathways involved in this regulation were not dissected in the present study, the combined *in vivo* and *in vitro* results support the view that NET-associated pathology is functionally involved in sleep deprivation-aggravated oral ulcers and can be effectively modulated by a localized biomaterial-based intervention.

From a materials perspective, Nio-MT@S-G MN combines several advantages in a single platform. The niosome component enhances melatonin encapsulation and provides a sustained release profile, while the SilMA/GelMA dual-network hydrogel endows the microneedles with suitable mechanical strength, biocompatibility and biodegradability. Notably, the S-G MN matrix alone also promoted HaCaT migration *in vitro*, suggesting that the hydrogel itself contributes to epithelial repair beyond serving as a delivery vehicle. This design allows painless penetration of the keratinized oral mucosa and localized delivery of melatonin directly into the lesion. Compared with conventional systemic or topical formulations (e.g., oral or spray administration), the microneedle system has the potential to reduce systemic exposure, increase local bioavailability and enable more precise modulation of the inflammatory microenvironment at the wound site.

In a mouse model combining sleep deprivation and chemically induced oral ulcers, Nio-MT@S-G MN accelerated ulcer closure, mitigated local inflammatory responses and partially restored the expression of barrier-related proteins. These findings indicate that the system can retain its reparative function in a complex pathological setting characterized by altered immune status. Sleep disorders often present as chronic and recurrent conditions. A localized delivery strategy that minimizes systemic burden and facilitates repeated administration may therefore offer practical advantages in clinical management.

Several limitations of this study should be acknowledged. First, the melatonin-neutrophil-NETs axis was not explored at the level of specific signaling pathways, and further work is needed to clarify how melatonin-loaded niosomes modulate neutrophil activation and NET formation. Second, while we hypothesize that the microneedle system may reduce systemic exposure compared to systemic administration, we did not directly measure melatonin concentrations in blood or major organs. This advantage remains theoretical and warrants pharmacokinetic validation in future studies. Third, the *in vitro* dissolution test was performed under static PBS conditions, which do not fully recapitulate the dynamic oral environment; *in vivo* dissolution kinetics may vary accordingly. Fourth, all animal experiments were performed exclusively on female C57BL/6 mice. While female mice were chosen to avoid inter-male aggression and to maintain consistency with our established protocol, we recognize that sex-dependent differences in immune responses, melatonin metabolism and wound healing may exist. Future studies incorporating both sexes are warranted to validate the translational relevance of our findings. Fifth, the modified multiple-platform water method, while widely used for sleep deprivation studies, inevitably introduces physical stress that could independently affect ulcer healing. Although this model was chosen for its advantage over single-platform designs in minimizing non-specific stress by allowing animals to move among multiple platforms, we acknowledge that some degree of physical stress is unavoidable. Future studies employing more refined behavioral or clinical models, as well as larger animal studies, will be required to better assess the translational potential of this approach.

Despite these limitations, this study integrates biomaterial design with pathological microenvironment regulation and proposes a NETs-focused microneedle delivery strategy for oral mucosal injury under sleep deprivation. The Nio-MT@S-G MN system illustrates how local immune modulation and barrier repair can be combined within a single regenerative platform and suggests that microneedle-based delivery may be extended to other chronic mucosal or cutaneous wounds characterized by dysregulated neutrophil responses.

## Conclusion

This study presents the Nio-MT@S-G MN as a localized therapeutic strategy for sleep deprivation-aggravated oral ulcers. By combining sustained transmucosal melatonin delivery with a bioactive composite microneedle matrix, Nio-MT@S-G MN effectively suppresses NET-associated inflammatory dysregulation, restores keratinocyte migratory and barrier functions and accelerates mucosal healing *in vivo*, while maintaining favorable mechanical properties and biosafety. Overall, these findings establish Nio-MT@S-G MN as a promising biomaterial-based platform that achieves localized melatonin delivery, suppresses NET-associated inflammation and restores epithelial barrier integrity, highlighting its potential for the treatment of NETs-associated refractory mucosal injuries.

## Supplementary Material

rbag180_Supplementary_Data

## Data Availability

The data that support the findings of this study are available from the corresponding author upon reasonable request.
