# Supplementary material for: Niosome-mediated melatonin delivery via SilMA/GelMA microneedles promotes healing of sleep deprivation-aggravated oral ulcers
Source: Regen Biomater. 2026 Aug 18;13:rbag180. doi: 10.1093/rb/rbag180 (PMC13637851; doi:10.1093/rb/rbag180)
Supplement: Supplementary file 1 [file rbag180_supplementary_data.zip › Response to reviewers.docx]

Dear editors and reviewers:

Thank you for your great efforts in reviewing our manuscript entitled “Niosome-mediated melatonin delivery via SilMA/GelMA microneedles promotes healing of sleep deprivation-aggravated oral ulcers” (manuscript ID: RB-2026-424.R1). We would like to thank you for allowing us to resubmit a revised copy of the manuscript and we highly appreciate your time and consideration.

These comments are all valuable and very helpful for revising and improving our manuscript, as well as the important guiding significance to our research. We have studied these comments carefully and have made corresponding corrections which we hope to meet with approval. Revised portions are highlighted in yellow in the manuscript for easy check/editing purpose. The following are point to point responses to reviewers’ comments.

**Responds to the Reviewers’ Comments:**

**Reviewer: 1**

**Major concerns**

**Comment 1:**

Page 24, line 45: The reduction in SYTOX Green signal (Fig. 4) may be due to reduced NET formation, but it could also result from decreased neutrophil viability. Please provide evidence to distinguish between these two possibilities.

**Response:**

We thank the reviewer for this important distinction. To address this concern, we performed CCK-8 assays on isolated mouse bone marrow neutrophils treated with Nio-MT@S-G MN extracts at concentrations corresponding to those used in the NETs suppression experiments (0.7, 1.37, 2.02, 2.65, and 3.25 μM).

Figure. S3

As shown in Fig. S3, neutrophil viability remained above 85% across all tested concentrations, with no significant differences compared to the untreated control group (p > 0.05 for all comparisons). The highest concentration (3.25 μM) showed approximately 85% viability, indicating that Nio-MT@S-G MN does not exert significant cytotoxicity on neutrophils under the experimental conditions employed.

**Page 24, line 45:**Functionally, immunofluorescence indicated diminished CitH3 deposition after treatment, and SYTOX Green staining together with flow cytometry showed reduced extracellular DNA release (Figure 4d-h). To rule out cytotoxicity as the cause of reduced SYTOX Green signal, CCK-8 assays confirmed that Nio-MT@S-G MN did not affect neutrophil viability at the tested concentrations **(Figure. S3)**.

**Comment 2:**

Page 27, line 14: The study only uses scratch, Transwell, and ZO‑1 immunofluorescence to evaluate migration. Please consider evaluating whether NETs induce apoptosis in HaCaT cells and whether Nio‑MT@S‑G MN alleviates this apoptosis.

**Response:**

We sincerely thank the reviewer for this valuable suggestion. We fully agree that excluding the possibility of apoptosis is important for interpreting the observed migration defects. To address this concern, we performed Annexin V-FITC/PI flow cytometry on HaCaT cells under the three conditions (CTR, NETs, and NETs + Nio-MT@S-G MN). As shown in Fig. S5, the percentages of viable cells (Q4) were 97.8%, 97.9%, and 97.5% for CTR, NETs, and NETs + Nio-MT@S-G MN groups, respectively, with no notable differences in early apoptotic (Q3) or late apoptotic/necrotic (Q2) populations among the groups. These results indicate that NETs at the concentration used in our study (5 μg/mL, 24 h) do not induce detectable apoptosis in HaCaT cells, and that Nio-MT@S-G MN treatment does not further affect cell viability.

Therefore, the impaired keratinocyte migration observed upon NETs exposure is primarily attributable to functional inhibition of cell motility and disruption of tight junctions rather than apoptosis induction. We have incorporated this finding into the revised manuscript.

Figure. S5

**Page 27, line 14:** Annexin V-FITC/PI flow cytometry confirmed that NETs did not induce apoptosis in HaCaT cells **(Figure. S5)**, indicating that the impaired migration is due to functional inhibition rather than cell death. Collectively, these results demonstrate that Nio-MT@S-G MN effectively preserves epithelial barrier integrity and mitigates NETs-mediated structural damage.

**Comment 3:**

Page 27, line 14: "exogenous NETs markedly impaired keratinocyte migration" but does not explain why the concentration of 5 μg/mL was selected. Please clarify whether this concentration is supported by literature, or provide preliminary experimental data that justify this choice.

**Response:**

We thank the reviewer for pointing out this important omission. We completely agree that the rationale for selecting the 5 μg/mL concentration of exogenous NETs should be clearly stated. We performed preliminary dose-response experiments (1, 2.5, 5, and 10 μg/mL) prior to the formal experiments to determine the optimal concentration. As shown in Fig. S2, 5 μg/mL achieved achieved substantial inhibition of HaCaT scratch closure (Fig. 5d-e) while maintaining cell viability above 85%, representing the optimal balance between functional inhibition and cell viability.

Figure. S2

**Page 14, line 56:** The concentration of exogenous NETs (5 μg/mL) was selected based on our preliminary dose-response experiments (1, 2.5, 5, and 10 μg/mL), in which 5 μg/mL achieved optimal inhibition of HaCaT migration without significant cytotoxicity **(Figure. S2)**.

**Minor concerns**

**Comment 1:**

Page 3, line 6: "However, Oral ulcers in the presence of sleep disturbance..."The "O" in "Oral" should not be capitalized. Please change to lowercase "oral". Page 3, line 12: In "and are highly prevalent", the second "are" can be omitted to avoid repetition. Page 3, line 45: "In the oral ulcer" is used as a general reference. Please revise to "In oral ulcers" or "In the context of oral ulcers". Page 4, line 33: "To improve local drug delivery in the oral mucosa" change "in" to "to" ("to the oral mucosa").

**Response:**

All of these grammatical corrections have been made in the revised manuscript. The changes are highlighted in the text.

**Page 3, line 6:** However, oral ulcers in the presence of sleep disturbance have been reported to exhibit delayed healing and aggravated inflammatory responses...

**Page 3, line 12:** Protein was isolated by SDS-PAGE and transferred to polyvinylidene fluoride membranes (Millipore, USA), blocked with 5% skim milk solution for 1.5 h.

**Page 3, line 45:** Sleep disorders are now recognized as a major global health challenge and highly prevalent among adults...

**Page 4, line 33:** In the context of oral ulcer, neutrophils are also central to mucosal immune homeostasis and have been increasingly implicated in oral inflammatory and ulcerative diseases

**Comment 2:**

Page 19, line 8: The encapsulation efficiency peaks at 1.2 mg/mL, but the drug loading still appears to increase at higher concentrations. Why was a higher concentration (e.g., 1.6 mg/mL) not selected to achieve greater drug loading? Please justify the choice of 1.2 mg/mL.

**Response:**

We thank the reviewer for this question. We chose 1.2 mg/mL because it represented the peak of encapsulation efficiency. Although drug loading continued to rise at higher concentrations, the encapsulation efficiency began to decline, indicating that excess drug was not efficiently incorporated into the niosomes. Therefore, 1.2 mg/mL was selected as the optimal balance between drug loading and encapsulation efficiency.

**Comment 3:**

Page 26, line 45: In Fig. 5a‑b, the Nio‑MT@S‑G MN group shows the highest scratch closure rate, but the S‑G MN alone group also shows higher closure than the control, suggesting the carrier itself may promote migration. Please discuss in the Discussion whether the GelMA/SilMA material has pro‑migratory activity, and provide literature support if available.

**Response:**We thank the reviewer for this careful observation. We fully agree that the S-G MN group exhibited a modest but consistent increase in HaCaT cell migration compared to the untreated control, suggesting intrinsic pro-migratory activity of the GelMA/SilMA composite hydrogel.Accordingly, we have added a paragraph in the Discussion section to address this point.

**Page 34, line 58:** while the SilMA/GelMA dual-network hydrogel endows the microneedles with suitable mechanical strength, biocompatibility, and biodegradability. Notably, the S-G MN matrix alone also promoted HaCaT migration in vitro, suggesting that the hydrogel itself contributes to epithelial repair beyond serving as a delivery vehicle.

**Comment 4:**

Page 6, line 51: Only female mice were used in all animal experiments. Please mention this as a limitation in the Discussion, explain the rationale for choosing females, and state that future studies should include males for validation.

**Response:**We thank the reviewer for this important consideration. We have added this limitation to the Discussion section as the fourth point in the limitations paragraph, acknowledging the exclusive use of female mice while explaining the rationale and indicating the need for future validation in males.

**Page 35, line 41:** Fourth, all animal experiments were performed exclusively on female C57BL/6 mice. While female mice were chosen to avoid inter-male aggression and to maintain consistency with our established protocol, we recognize that sex-dependent differences in immune responses, melatonin metabolism, and wound healing may exist. Future studies incorporating both sexes are warranted to validate the translational relevance of our findings.

**Comment 5:**

Page 35, line 6: The manuscript states that the microneedle system can "reduce systemic exposure", but this study did not measure melatonin concentrations in the blood or distribution in major organs. Please acknowledge in the Discussion that this is a theoretical advantage not directly measured here..

**Response:**We thank the reviewer for this important point. We have revised the relevant text to acknowledge this limitation.

**Page 35, line 41:**Second, while we hypothesize that the microneedle system may reduce systemic exposure compared to systemic administration, we did not directly measure melatonin concentrations in blood or major organs. This advantage remains theoretical and warrants pharmacokinetic validation in future studies.

**Comment 6:**

Page 36, line 25: "integrates immune microenvironment modulation with epithelial regeneration"please rephrase to a more specific summary.

**Response:**We thank the reviewer for this suggestion. We have revised the sentence to more specifically summarize our findings.

**Page 36, line 25:** Overall, these findings establish Nio-MT@S-G MN as a promising biomaterial-based platform that achieves localized melatonin delivery, suppresses NETs-associated inflammation, and restores epithelial barrier integrity, highlighting its potential for the treatment of NETs-associated refractory mucosal injuries.

**Reviewer: 2**

**Comment 1:**

Page 42, line 51, the manuscript is submitted to Regenerative biomaterials, but the author mentioned “We believe that this manuscript is well suited for publication in Advanced Healthcare Materials”. This is a very elementary mistake

**Response:**

We sincerely apologize for this careless error. This was an oversight on our part during the final preparation of the manuscript, and we are truly embarrassed by this mistake. We have removed the incorrect sentence and have carefully reviewed the entire manuscript and cover letter to ensure they are now appropriately aligned with the scope of Regenerative Biomaterials. We are grateful to the reviewer for catching this, and we will take extra care in future submissions to avoid such errors.

**Page 42, line 51:** We believe that this manuscript is well suited for publication in Regenerative Biomaterials because it combines biomaterials design, local drug delivery, immune regulation, and mucosal repair, which are closely aligned with the journal’s scope and readership.

**Comment 2:**

The manuscript demonstrates that the microneedle system can inhibit the release of eutrophil extracellular traps (NETs) and reduce the expression of PAD4 and CitH3.However, how exactly does melatonin (MT) inhibit NETs? There is no explanation for this at all in the text.

**Response:**

We sincerely thank the reviewer for pointing out this critical mechanistic gap. We have added a mechanistic discussion of melatonin's NET-inhibitory effects in the Discussion section.

**Page 34, line 14:** On this basis, targeting NETs-associated pathology appears to be a rational therapeutic strategy for this underrecognized condition. Melatonin was selected as the active agent for this purpose based on its well-established capacity to counteract NETs formation. Mechanistically, melatonin suppresses NETs through convergent pathways: it scavenges reactive oxygen species (ROS), intercepting the upstream oxidative trigger of NETosis^[57]^, and inhibits the NF-κB signaling pathway, reducing the pro-inflammatory transcriptional program that primes neutrophils for NETs formation^[58]^. These mechanisms converge to suppress the PAD4/CitH3 axis and reduce extracellular DNA release, consistent with the reductions observed in our study. Accordingly, we developed a melatonin-loaded niosome–hydrogel microneedle system (Nio-MT@S-G MN) to locally modulate the pathological microenvironment of sleep deprivation-aggravated oral ulcers.

59	Wang, B., et al., *Melatonin alleviates rheumatoid arthritis via elimination of damaged mitochondria.* Autophagy, 2026(just-accepted).

60.	Wu, X., et al., *Melatonin Alleviates Periodontitis by Suppressing Neutrophil Extracellular Traps–Dependent Neutrophil Recruitment.* Journal of Clinical Periodontology, 2026. **53**(4): p. 630-643.

**Comment 3:**

There are a large number of cases where abbreviations and full names are mixed in the text, which greatly affects the readability and scientific rigor of the article.

**Response:**

We sincerely apologize for this oversight. We fully agree that consistent use of abbreviations is essential for clarity and scientific rigor. We have now carefully reviewed the entire manuscript and standardized all abbreviations throughout. Each abbreviation is now defined at its first appearance in the Abstract, Introduction, and main text. A complete list of abbreviations has also been provided in the Supplementary Information (Table S1) for easy reference.

We are grateful to the reviewer for bringing this to our attention, and we trust that the revised manuscript now meets the expected standards of readability.

| **Abbreviation** | **Full term** |
| --- | --- |
| Biological terms | |
| NETs | Neutrophil extracellular traps |
| RBCs | Red blood cells |
| PAD4 | Peptidylarginine deiminase 4 |
| CitH3 | Citrullinated histone H3 |
| MPO | Myeloperoxidase |
| NE | Neutrophil elastase |
| IL-1β | Interleukin-1β |
| TNF-α | Tumor necrosis factor-α |
| ZO-1 | Zonula occludens-1 |
| Experimental groups | |
| OU | Oral ulcer under normal sleep conditions |
| OUS | Oral ulcer with sleep deprivation |
| Materials & formulations | |
| hematoxylin and eosin | HE |
| SilMA | Silk fibroin methacrylate |
| GelMA | Gelatin methacryloyl |
| Nio-MT@S-G MN | Melatonin-loaded niosome-incorporated SilMA/GelMA microneedle |
| Nio | Blank niosome |
| Nio-MT | Melatonin-loaded niosome |
| S-G MN | Blank SilMA/GelMA microneedle |
| Nio@S-G MN | Blank niosome-loaded SilMA/GelMA microneedle |
| Characterization & assays | |
| TEM | Transmission electron microscopy |
| SEM | Scanning electron microscopy |
| FTIR | Fourier transform infrared spectroscopy |
| CCK-8 | Cell Counting Kit-8 |
| RT-qPCR | Reverse transcription‑quantitative real‑time PCR |
| Other | |
| DMSO | Dimethyl sulfoxide |
| FBS | Fetal bovine serum |
| EER | Encapsulation efficiency rate |
| DLR | Drug loading rate |

**Comment 4:**

Similarly, sleep deprivation is a systemic neuroendocrine disorder. How it "pre-stimulates" neutrophils in the bone marrow is one of the core points of this manuscript, but it is not elaborated in depth in the text

**Response:**

We sincerely thank the reviewer for this critical and insightful comment. We fully agree that the mechanistic link between sleep deprivation and bone marrow neutrophil priming is central to our study, and we regret that this was not adequately elaborated in the original manuscript. Based on the landmark work by McAlpine et al., we have now added a detailed mechanistic discussion in the Introduction section.

**Page 34, line 14:** Given that sleep deprivation has been reported to aggravate oral ulcer inflammation and delay healing, and that the underlying mechanism may involve neuro-immune-endocrine dysregulation^[4, 18]^, it is important to consider how sleep loss shapes neutrophil biology beyond the peripheral circulation. Recent evidence indicates that sleep deprivation fundamentally reprograms the bone marrow, where neutrophils originate, to produce cells that are intrinsically primed for heightened inflammatory responses. McAlpine et al. demonstrated that sleep fragmentation reduces hypothalamic hypocretin (orexin) production, which normally restricts CSF1 production by pre-neutrophils in the bone marrow. Sleep deprivation relieves this restraint, leading to elevated CSF1 levels that drive myeloid-biased hematopoiesis and the generation of neutrophils that are more responsive to subsequent inflammatory stimuli^[19]^. Consistent with this, sleep-deprived rats exhibit mild regenerative neutrophilia accompanied by immature neutrophils, and sleep-deprived medical residents show altered neutrophil function^[17, 18]^. Thus, neutrophils activated under sleep-deprived conditions may be particularly prone to release NETs at sites of tissue injury, contributing to aggravated inflammation and delayed healing in oral ulcers. This positions neutrophils and their effector functions—particularly NETs formation—as rational therapeutic targets for sleep disturbance-associated oral mucosal pathologies.

19.	McAlpine, C.S., et al., Sleep modulates haematopoiesis and protects against atherosclerosis. Nature, 2019. 566(7744): p. 383-387.

**Comment 5:**

The author mentioned that the microneedle tips dissolved within 4 minutes in static PBS. The oral environment is a highly dynamic one filled with saliva flushing. If the microneedle matrix disintegrates within 4 minutes, the nano-lipids will be immediately exposed to saliva and rapidly swallowed or diluted, making it impossible to achieve the local long-term retention and continuous release claimed at the beginning of the article.

**Response:**

We thank the reviewer for this important clarification. We would like to clarify that in our system, the microneedle matrix and the niosomes serve distinct and complementary roles. The SilMA/GelMA matrix is designed to rapidly disintegrate simply to deliver the niosome payload into the subepithelial tissue. Once deposited there, the niosomes-not the matrix-provide the sustained release (approximately 180 minutes, as shown in Fig. 2h). Unlike surface-applied formulations that remain on the mucosa and are directly exposed to saliva, our microneedles penetrate the epithelial barrier, so the niosomes are embedded within the tissue and protected from salivary clearance.

**Comment 6:**

FTIR of SilMA and GelMA both showed amide I–III and amide A bands peaks. The author's empty assertion that the formation of β-sheet structures of silk fibroin within the hydrogel matrix is not rigorous. The horizontal coordinate of the figure is also incorrect.

**Response:**

We sincerely thank the reviewer for pointing out this lack of rigor. We agree that our previous interpretation of the FTIR data was insufficiently detailed, and we acknowledge that the horizontal coordinate of the figure was incorrectly labeled. In the revised manuscript, we have re-analyzed the FTIR spectra with specific peak assignments and have included supporting literature to substantiate the assignment of β-sheet structures. The horizontal axis of Figure 2I has been corrected to Wavenumber (cm⁻¹).

Figure 2I

**Page 19, line 25:** FTIR analysis of the SilMA/GelMA composite hydrogel revealed characteristic absorption bands at 1631.08 cm⁻¹ (amide I, C=O stretching), 1520.18 cm⁻¹ (amide II, N-H bending), 1237.45 cm⁻¹ (amide III, C-N stretching), and 3275.83 cm⁻¹ (amide A, N-H stretching). The amide I band at 1631.08 cm⁻¹ falls within the characteristic range for β-sheet conformation of silk fibroin (1620–1640 cm⁻¹), while the amide A band confirms the presence of hydrogen bonding interactions^[54]^. These spectral features indicate the successful incorporation of silk fibroin-based components and the preservation of β-sheet secondary structures **(Figure 2l)**.

54.	Kim, S.H., et al., Precisely printable and biocompatible silk fibroin bioink for digital light processing 3D printing. Nat Commun, 2018. 9(1): p. 1620.

**Comment 7:**

In the in vivo experiments, the authors directly leaped from the OUS group to the OUS+ microneedle treatment group. This can only prove that microneedles are effective, but it cannot be 100% proven in vivo that microneedles promote healing by eliminating/inhibiting NETs. An additional animal control group can be added: local application of PAD4 inhibitors in the OUS group of mice can fully prove it if the effect is consistent with that of the treatment group

**Response:**

We sincerely thank the reviewer for this critical and constructive suggestion. We fully agree with the reviewer that the original three-group comparison (OU, OUS, OUS+Nio-MT@S-G MN) was insufficient to establish a causal link between NETs inhibition and therapeutic efficacy in vivo. To address this concern, we performed an additional experiment including Cl-amidine, a specific PAD4 inhibitor that blocks NETs formation at the level of histone citrullination, as a positive control group in the OUS model. Cl-amidine (50 mg/kg) was administered via intraperitoneal injection . As shown in the updated Figure. S6 the Cl-amidine-treated group exhibited: 1. Accelerated wound closure, with a healing trajectory nearly superimposable on that of the Nio-MT@S-G MN-treated group, and significantly superior to the OUS group. 2. Markedly reduced CitH3 protein expression in ulcer tissues (Western blot), to levels comparable to those achieved by Nio-MT@S-G MN treatment.

Together, these findings provide direct in vivo evidence that pharmacological NETs inhibition (via PAD4 blockade) is sufficient to recapitulate the therapeutic benefits of Nio-MT@S-G MN. This establishes a causal relationship between NETs suppression and accelerated ulcer healing, confirming that the therapeutic efficacy of our microneedle system is predominantly mediated through inhibition of aberrant NETs formation.

We have incorporated these datas into the Supporting information. We are deeply grateful to the reviewer for prompting us to provide this critical mechanistic validation.

Figure. S6

**Page 31, line 5:** To verify the NET-dependent mechanism, we included the PAD4 inhibitor Cl-amidine as a positive control **(Figure. S6)**, which reduced CitH3/PAD4 expression and accelerated wound closure, recapitulating the effects of Nio-MT@S-G MN.

**Comment 8:**

The number of the control group was insufficient, making it impossible to break down the contribution: whether it was ** free melatonin or ** nanocarriers that played a role in inhibiting NETs and promoting migration.Or does the GelMA/SilMA degradation product itself also have a weak effect?

**Response:**

We sincerely thank the reviewer for this important point. We fully agree that the original three-group comparison (OU, OUS, OUS+Nio-MT@S-G MN) was insufficient to deconvolute the individual contributions of each component in vivo. To address this, we performed an additional animal experiment with the OUS,OUS+Melatonin, OUS+Niosome, OUS+S-G MN and OUS+Nio-MT@S-G MN group.

Figure. S7

Furthermore, control experiments with individual components (free melatonin, blank niosomes, and blank S-G microneedles) confirmed that the full integrated system is required for therapeutic efficacy **(Figure. S7)**.

**Reviewer: 3**

**Comment 1:**

The selection of 5 μg/mL exogenous NETs lacks justification. Please specify whether this concentration is supported by literature or by preliminary experimental evidence.

**Response:**

We thank the reviewer for pointing out this important omission. We completely agree that the rationale for selecting the 5 μg/mL concentration of exogenous NETs should be clearly stated. We performed preliminary dose-response experiments (1, 2.5, 5, and 10 μg/mL) prior to the formal experiments to determine the optimal concentration. As shown in Fig. S2 5 μg/mL achieved achieved substantial inhibition of HaCaT scratch closure (Fig. 5d-e) while maintaining cell viability above 85%, representing the optimal balance between functional inhibition and cell viability.

Figure. S2

**Page 14, line 56:** The concentration of exogenous NETs (5 μg/mL) was selected based on our preliminary dose-response experiments (1, 2.5, 5, and 10 μg/mL), in which 5 μg/mL achieved optimal inhibition of HaCaT migration without significant cytotoxicity **(Figure. S2)**.

**Comment 2:**

Quantitative protein-level evidence for ZO-1 is recommended to support the immunofluorescence findings.

**Response:**

We thank the reviewer for this valuable suggestion. We have performed Western blot analysis of ZO-1 expression in HaCaT cells under the three conditions (CTR, NETs, and NETs + Nio-MT@S-G MN). To maintain the focus of the main text on the key findings, we have placed the detailed data in the Supplementary Information (Figure. S4). In the main text, we have briefly summarized the key result.

Figure. S4

**Page 27, line 12:** Western blot analysis further confirmed that NETs exposure reduced ZO-1 protein expression, which was restored upon Nio-MT@S-G MN treatment **(Figure. S4).**

**Comment 3:**

In the discussion section: The in vitro dissolution test shows that microneedles dissolve completely within 4 minutes in static PBS. However, the oral cavity is a dynamic environment with saliva flow, tongue movement, and mechanical friction, which may alter dissolution kinetics.

**Response:**

We thank the reviewer for this important consideration. We fully agree that the oral cavity is a dynamic environment that differs from static in vitro conditions.

We would like to clarify that the 4-minute dissolution time reflects the rapid disintegration of the microneedle matrix, which is an intentional design feature. The matrix serves primarily as a mechanical vehicle to penetrate the epithelial barrier and deposit the niosome payload directly into the subepithelial tissue. Once deposited, the niosomes—not the matrix—provide sustained melatonin release (~180 min, Fig. 2h), protected from salivary clearance by the tissue itself. This two-stage design differs fundamentally from surface-applied formulations that remain exposed to saliva.

We acknowledge that in vivo dissolution kinetics may differ from in vitro conditions, and have added a brief statement in the Discussion section to address this.

**Page 35, line 41:** Fifth, the in vitro dissolution test was performed under static PBS conditions, which do not fully recapitulate the dynamic oral environment; in vivo dissolution kinetics may vary accordingly.

**Comment 4:**

Please add a sentence in the Discussion acknowledging this difference and noting that in vivo dissolution behavior may vary.

**Response:**

We thank the reviewer for this important consideration. We fully agree that the oral cavity is a dynamic environment that differs from static in vitro conditions.

We acknowledge that in vivo dissolution kinetics may differ from in vitro conditions, and have added a brief statement in the Discussion section to address this.

**Page 35, line 41:** Fifth, the in vitro dissolution test was performed under static PBS conditions, which do not fully recapitulate the dynamic oral environment; in vivo dissolution kinetics may vary accordingly.

**Comment 5:**

All animal experiments were performed on female mice only. Please acknowledge this as a limitation, justify the use of females.

**Response:**

We thank the reviewer for this important consideration. We have added this limitation to the Discussion section as the fourth point in the limitations paragraph, acknowledging the exclusive use of female mice while explaining the rationale and indicating the need for future validation in males.

**Page 35, line 41:** Fourth, all animal experiments were performed exclusively on female C57BL/6 mice. While female mice were chosen to avoid inter-male aggression and to maintain consistency with our established protocol, we recognize that sex-dependent differences in immune responses, melatonin metabolism, and wound healing may exist. Future studies incorporating both sexes are warranted to validate the translational relevance of our findings.

**Comment 6:**

The modified multiple-platform water method, while widely used, inevitably introduces physical stress that could independently affect ulcer healing. Please discuss this limitation in the Discussion.

**Response:**

We sincerely thank the reviewer for this important methodological consideration. We fully agree that the modified multiple-platform water method, while widely used for sleep deprivation studies, inevitably introduces physical stress (intermittent water, restricted mobility, and social isolation) that could independently influence inflammatory status and wound healing. We have added a brief statement acknowledging this limitation in the Discussion section (Limitations paragraph).

We note that this method was chosen over the single-platform model because it minimizes non‑specific stress by allowing animals to move among multiple platforms, thereby reducing the physical confinement associated with single‑platform designs. Nevertheless, we agree that some degree of physical stress is unavoidable, and we have acknowledged this as a limitation in the revised manuscript.

**Page 35, line 41:** Fifth, the modified multiple-platform water method, while widely used for sleep deprivation studies, inevitably introduces physical stress that could independently affect ulcer healing. Although this model was chosen for its advantage over single-platform designs in minimizing non-specific stress by allowing animals to move among multiple platforms, we acknowledge that some degree of physical stress is unavoidable.

**Comment 7:**

Chloroform is used to dissolve lipids before rotary evaporation to form a thin film. Was chloroform completely removed? If removal was performed, please describe the method.

**Response:**

We thank the reviewer for this question. Chloroform was removed by rotary evaporation at 30°C for 30 min under 200 mbar pressure, followed by further drying under reduced pressure at room temperature for an additional 12 h in a vacuum desiccator. This is a standard and widely used procedure for niosome preparation, and the extended vacuum drying ensures complete solvent removal. We have added this description to the Methods section

**Page 8, line 4:** The thin film was obtained by evaporating the mixture solution at 30°C for 30min under 200 mbar pressure on a rotary evaporator (Yarong, China), followed by further drying under reduced pressure at room temperature for 12 h in a vacuum desiccator.

Minor revision:

**Comment 1:**

Regarding the title: The subject of the sentence is “delivery” (singular), but the verb “promote” is plural (should be “promotes”). Please correct this grammatical error.

**Response:**

We sincerely thank the reviewer for catching this grammatical error. We have corrected the title as suggested.

**Page 1, line 3:** Niosome-mediated melatonin delivery via SilMA/GelMA microneedles promotes healing of sleep deprivation-aggravated oral ulcers

**Comment 2:**

In Introduction section, it contains frequent passive voice constructions (e.g., “have been reported”, “are now recognized”). Please consider revising some of these to active voice to improve readability.

**Response:**

We thank the reviewer for this helpful suggestion. We have revised the Introduction to incorporate more active voice constructions where appropriate, and have eliminated redundant passive expressions to improve readability.

**Page 4, line 33:** To improve local drug delivery to the oral mucosa, researchers have explored a variety of material platforms, including mucoadhesive patches, hydrogels, and microneedle systems^[25-29]^.

**Comment 3:**

The first paragraph of Introduction ends by stating that no specific therapy exists for sleep deprivation‑aggravated oral ulcers. The second paragraph then jumps directly into neutrophils and NETs. A transitional sentence explaining why neutrophils were selected as a candidate target is missing. Please add a brief sentence to bridge this gap.

**Response:**

We thank the reviewer for this suggestion. We have added a brief transitional sentence at the beginning of the second paragraph to bridge the gap.

**Page 3, line 29:** Neutrophils are central to both oral mucosal inflammation and sleep‑related immune dysregulation. As the most abundant innate immune cells, neutrophils serve as early responders during tissue injury, contributing to antimicrobial defense, inflammatory orchestration, and wound repair through phagocytosis, degranulation, reactive oxygen species release, and NETs formation^[9-13]^.

**Comment 4:**

The third paragraph of Introduction, the two points regarding “drug dilution by saliva” and “short local exposure” are essentially the same. Please merge them into a single point to avoid repetition.

**Response:**

We thank the reviewer for this suggestion. We agree that the two points regarding drug dilution and short local exposure are essentially the same. We have merged them into a single point in the third paragraph of the Introduction and removed the redundant sentence on short local exposure. The change has been highlighted in the revised manuscript.

**Page 4, line 6:** First, drugs applied to the oral mucosa are readily diluted and cleared by saliva and mechanical activities, resulting in poor local retention and limited therapeutic persistence^[23, 24]^.

**Comment 5:**

The last paragraph of Introduction, the listed properties of melatonin are well known. Please simplify to: “melatonin was chosen for its known anti-inflammatory and immunomodulatory properties.”

**Response:**

We thank the reviewer for this suggestion. We have simplified the description of melatonin's properties in the last paragraph of the Introduction.

**Page 5, line 12:** melatonin was selected for its well‑established antioxidant and immunomodulatory properties, which are relevant to neutrophil‑associated inflammatory pathology.

**Comment 6:**

Please cite Figure 1 at the first mention of Nio-MT@S-G MN (e.g., “...composite hydrogel microneedle (Nio-MT@S-G MN, Figure 1)”) rather than at the end of the sentence.

**Response:**

We thank the reviewer for this suggestion. We have revised the text to cite Figure 1 at the first mention of Nio-MT@S-G MN in the Introduction, rather than at the end of the sentence.

**Page 5, line 4:** Therefore, we constructed the Nio-MT@S-G MN for localized transmucosal delivery and sustained release **(Figure 1)**.

**Comment 7:**

In results section, description of microneedle dissolution (Fig. 2m): The phrase “rapidly swelled and progressively dissolved” is somewhat contradictory. Please rephrase for clarity.

**Response:**

We thank the reviewer for pointing out this unclear description. We have rephrased the sentence to more accurately and clearly describe the dissolution behavior.

**Page 19, line 29:** In a PBS degradation assay, the microneedle tips rapidly disintegrated upon contact with PBS, with nearly complete dissolution observed within 4 min (Fig. 2m)

**Comment 8:**

In discussion section, the last sentence of the fourth paragraph is overly long. Please split it into two shorter sentences.

**Response:**

We thank the reviewer for this suggestion. We have split the sentence into two shorter sentences for improved readability.

**Page 35, line 21:** Sleep disorders often present as chronic and recurrent conditions. A localized delivery strategy that minimizes systemic burden and facilitates repeated administration may therefore offer practical advantages in clinical management.

**Comment 9:**

the Conclusion mixes present and past tenses (e.g., “presents” vs. “suppressed”). Please unify the tense throughout the paragraph.

**Response:**

We thank the reviewer for this careful observation. We have unified the tense throughout the Conclusion section to the present tense, as it summarizes established findings. All past-tense verbs have been changed to present tense accordingly.

**Page 36, line 12:** This study presents the Nio-MT@S-G MN as a localized therapeutic strategy for sleep deprivation-aggravated oral ulcers. By combining sustained transmucosal melatonin delivery with a bioactive composite microneedle matrix, Nio-MT@S-G MN effectively suppresses NETs-associated inflammatory dysregulation, restores keratinocyte migratory and barrier functions, and accelerates mucosal healing in vivo, while maintaining favorable mechanical properties and biosafety. Overall, these findings establish Nio-MT@S-G MN as a promising biomaterial-based platform that achieves localized melatonin delivery, suppresses NETs-associated inflammation, and restores epithelial barrier integrity, highlighting its potential for the treatment of NETs-associated refractory mucosal injuries.

**Reviewer: 4**

**Comment 1:**

Immunofluorescence (Fig. 5h) suggests reduced ZO‑1 after NET exposure. To provide quantitative protein‑level support, please perform Western blot for ZO‑1 in HaCaT cells under the same three conditions.

**Response:**

We thank the reviewer for this valuable suggestion. We have performed Western blot analysis of ZO-1 expression in HaCaT cells under the three conditions (CTR, NETs, and NETs + Nio-MT@S-G MN). To maintain the focus of the main text on the key findings, we have placed the detailed data in the Supplementary Information (Figure. S4). In the main text, we have briefly summarized the key result.

Figure. S4

**Page 27, line 12:** Western blot analysis further confirmed that NETs exposure reduced ZO-1 protein expression, which was restored upon Nio-MT@S-G MN treatment **(Figure. S4)**.

**Comment 2:**

The study focuses on epithelial closure but does not assess collagen deposition or tissue remodeling in the healed mucosa. Please perform Masson’s trichrome staining on Day 6 ulcer tissue sections to evaluate the quality of wound healing.

**Response:**

We sincerely thank the reviewer for this valuable suggestion. We agree that assessing the quality of wound healing beyond epithelial closure is important. We have now performed Masson's trichrome staining on Day 6 ulcer tissue sections to evaluate collagen deposition and tissue remodeling in the healed mucosa.

Figure 6g

**Page 30, line 45:** Histological, histochemical and immunofluorescence analyses yielded consistent results **(Figure 6g)**. Severe epithelial disruption together with pronounced inflammatory cell infiltration was observed in the OUS group by H&E staining, whereas improved mucosal continuity and reduced inflammatory infiltration were evident after Nio-MT@S-G MN treatment. Masson's trichrome staining further revealed that collagen deposition in the healed mucosa was markedly reduced and disorganized in the OUS group, while Nio-MT@S-G MN treatment significantly restored collagen content and fibrillar organization, indicating improved tissue remodeling quality.

**Comment 3:**

The abstract is dense. Consider splitting the last sentence of the results section into two for readability.

**Response:**

We thank the reviewer for this suggestion. We have split the last sentence of the results section in the Abstract into two shorter sentences for improved readability.

**Page 2, line 47:** By integrating controlled drug delivery with immune microenvironment regulation, this microneedle provides a translational strategy for treating sleep disorder-aggravated mucosal injuries. It may also be applicable to other chronic wounds characterized by dysregulated neutrophil responses.

**Comment 4:**

Figure 2 subheading:Please add “the” before “construction/characterization” for clarity.

**Response:**

We thank the reviewer for this correction. We have revised the subheading of Figure 2 as suggested.

**Page 20, line 53:** Figure 2. Identification of neutrophil-associated pathology in sleep deprivation-aggravated oral ulcers and the construction/characterization of Nio-MT@S-G MN.

**Comment 5:**

“4 mg Span60, 8 mg cholesterol (ratio 3:1) and melatonin of different qualities (4,6,8,10 mg) was dissolved in 5 mL of chloroform.”“qualities” should be changed to “quantities” or “concentrations.” And “was” should be “were.”

**Response:**

We thank the reviewer for catching these errors. We have corrected the sentence as suggested.

**Page 7, line 54:** 24 mg Span60, 8 mg cholesterol (ratio 3:1) and melatonin of different quantities (4,6,8,10 mg) were dissolved in 5 mL of chloroform.

**Comment 6:**

Please provide a rationale for choosing 5 μg/mL of exogenous NETs.

**Response:**

We thank the reviewer for pointing out this important omission. We completely agree that the rationale for selecting the 5 μg/mL concentration of exogenous NETs should be clearly stated. We performed preliminary dose-response experiments (1, 2.5, 5, and 10 μg/mL) prior to the formal experiments to determine the optimal concentration. As shown in Fig. S2, 5 μg/mL achieved achieved substantial inhibition of HaCaT scratch closure (Fig. 5d-e) while maintaining cell viability above 85%, representing the optimal balance between functional inhibition and cell viability.

Figure. S2

**Page 14, line 56:** The concentration of exogenous NETs (5 μg/mL) was selected based on our preliminary dose-response experiments (1, 2.5, 5, and 10 μg/mL), in which 5 μg/mL achieved optimal inhibition of HaCaT migration without significant cytotoxicity **(Figure. S2)**.

**Comment 7:**

HaCaT cells are an immortalized cell line. Please specify the passage number limit used.

**Response:**

We thank the reviewer for this suggestion. We have now specified the passage number limit for HaCaT cells in the Methods section. In our experiments, HaCaT cells were used between passages 3 and 15 to ensure genetic and phenotypic stability.

**Page 12, line 6:** HaCaT cells were used between passages 3 and 15 for all experiments.

**Comment 8:**

The effect of Nio-MT@S-G MN on NETs is strong. However, to rule out a direct cytotoxic effect on neutrophils (reducing NETs by killing cells), add a brief viability check of neutrophils after treatment-can be mentioned in text or supplemental.

**Response:**

We sincerely thank the reviewer for this important control. To rule out the possibility that the reduced NETs formation was due to neutrophil cytotoxicity rather than specific inhibition of NETs we performed CCK‑8 viability assays on isolated mouse bone marrow neutrophils treated with Nio‑MT@S‑G MN extracts at concentrations corresponding to those used in the NETs suppression experiments.

Figure. S3

As shown in Fig. S3, neutrophil viability remained above 85% across all tested concentrations, with no significant differences compared to the untreated control group (p > 0.05 for all comparisons). The highest concentration (3.25 μM) showed approximately 85% viability, indicating that Nio-MT@S-G MN does not exert significant cytotoxicity on neutrophils under the experimental conditions employed.

**Page 24, line 45:** Functionally, immunofluorescence indicated diminished CitH3 deposition after treatment, and SYTOX Green staining together with flow cytometry showed reduced extracellular DNA release (Figure 4d-h). To rule out cytotoxicity as the cause of reduced SYTOX Green signal, CCK-8 assays confirmed that Nio-MT@S-G MN did not affect neutrophil viability at the tested concentrations **(Figure. S3)**.

**Comment 9:**

The phenol-induced ulcer model is standard, but specify the concentration of phenol (e.g., 80%? 100%?) for reproducibility.

**Response:**

We thank the reviewer for this suggestion. We have now specified the phenol concentration in the Methods section. In our experiments, 90% phenol was used to induce oral ulcers, as this concentration is widely adopted in the literature for establishing oral ulcer models in rats and mice.

**Page 6, line 58:** Oral ulcer induction: Mice were anesthetized by intraperitoneal injection of 0.3% sodium pentobarbital (0.15 mL per 10 g body weight, equivalent to 45 mg/kg)^[47]^. A cotton pellet (3 mm outer diameter, 2 mm inner diameter) soaked in 90% phenol was applied to the bilateral buccal mucosa for 45 s on each side to induce uniform ulcers^[48]^.

48.	Miao, M., et al., *Effect of Shuangjinlian mixture on oral ulcer model in rat.* Saudi J Biol Sci, 2019. **26**(4): p. 790-794.

**Comment 10:**

Chloroform is toxic; mention that residual solvent was removed (e.g., under vacuum) before adding water.

**Response:**

We thank the reviewer for this question. Chloroform was removed by rotary evaporation at 30°C for 30 min under 200 mbar pressure, followed by further drying under reduced pressure at room temperature for an additional 12 h in a vacuum desiccator. This is a standard and widely used procedure for niosome preparation, and the extended vacuum drying ensures complete solvent removal. We have added this description to the Methods section

**Page 8, line 4:** The thin film was obtained by evaporating the mixture solution at 30°C for 30min under 200 mbar pressure on a rotary evaporator (Yarong, China), followed by further drying under reduced pressure at room temperature for 12 h in a vacuum desiccator.

**Comment 11:**

UV crosslinking time and intensity are given, but power density (mW/cm²) is missing - add if available.

We thank the reviewer for this suggestion. We have now specified the UV power density in the Methods section.

**Page 10, line 30:** After centrifugation, the mold was exposed to UV light (405 nm, 8 mW/cm²) for 30 s to induce photocrosslinking.

**Comment 12:**

Statistical Analysis: Clearly state that normality tests (e.g., Shapiro-Wilk) were performed before choosing parametric tests.

We thank the reviewer for this suggestion. We have now clarified in the Statistical Analysis section that normality was assessed using the Shapiro-Wilk test prior to selecting parametric or nonparametric tests.

**Page 36, line 39:** Normality of data distribution was assessed using the Shapiro-Wilk test prior to statistical analysis.

We tried our best to improve the manuscript and made a number of changes in the manuscript. These changes will not influence the content and framework of the paper. And here, we do not list certain word corrections, but rather highlight them in yellow in the revised paper. We appreciate your warm work earnestly and hope that the correction will meet with approval. Once again, thank you very much for your comments and suggestions.

Thank you and best regards.

Sincerely yours,

Yuzhou Li D.D.S.

School and Hospital of Stomatology

Chongqing Medical University

426 Songshi North Road, Yubei District

Chongqing, P. R. of China. 401147

E-mail:

Yuzhou Li, 501182@cqmu.edu.cn.

Tel: +86-023-88860086

Fax: +86-23-8860-2351
