# Supplementary material for: Niosome-mediated melatonin delivery via SilMA/GelMA microneedles promotes healing of sleep deprivation-aggravated oral ulcers
Source: Regen Biomater. 2026 Aug 18;13:rbag180. doi: 10.1093/rb/rbag180 (PMC13637851; doi:10.1093/rb/rbag180)
Supplement: Supplementary file 1 [file rbag180_supplementary_data.zip › Supporting Information.docx]

Supporting Information

**Niosome-mediated melatonin delivery via SilMA/GelMA microneedles promotes healing of sleep deprivation-aggravated oral ulcers**

Ying Xie^#^, Qi Huang^#^, Xinzhi Jiang, Kehao Liu, Kamoran Tuerhong, Chengji Shi, Renjie Tang, Xuehui Hu, Youchun Zhang, Xiang Li, Mingcong Yang, Yuting He, Haixia Dang, Ming Wang, DanFeng Shen, Sheng Yang,Peng Yu^*^, Yuzhou Li^*^.

**Figure S1. Dose screening of individual components and composite formulations by CCK-8 assay.**

A) Cell viability of HaCaT cells after 24 h treatment with blank niosomes (Nio) at 0, 2, 4, 6, 8, and 10 μL per well. B) Cell viability of HaCaT cells after 24 h treatment with blank niosomes (Nio) at 0, 0.4, 0.8, 1.2, 1.6, and 2.0 μL per well. C) Cell viability of HaCaT cells after 24 h treatment with blank SilMA/GelMA microneedle extract (S-G MN) at final total polymer concentrations of 0.294, 0.577, 0.849, 1.111, and 1.364 mg mL⁻¹. D) Cell viability of HaCaT cells after 24 h treatment with melatonin-loaded niosomes (Nio-MT) at final concentrations of 0, 59.51, 119.02, 178.53, 238.04, and 297.55 μM. E) Cell viability of HaCaT cells after 24 h treatment with melatonin-loaded niosomes (Nio-MT) at final concentrations of 0, 23.804, 47.608, 71.412, 95.216, and 119.02 μM. F) Cell viability of HaCaT cells after 24 h treatment with blank niosome-loaded S-G microneedle extract (Nio@S-G MN) at 0, 2, 4, 6, 8, and 10 μL per well. Data are presented as mean ± SD. Statistical significance was analyzed as described in the Methods section. *P < 0.05, **P < 0.01, ***P < 0.001, ****P < 0.0001; ns, not significant.

**Figure S2. Cytotoxicity evaluation of exogenous NETs on HaCaT cells.**

HaCaT cells were treated with increasing concentrations of NETs (1.25-10 μg/mL) for 24 h. Cell viability remained above 95% at all tested concentrations, as determined by CCK‑8 assay. Data are mean ± SD from three independent experiments.

**Figure S3. Neutrophil viability after Nio-MT@S-G MN treatment.**

Neutrophils were treated with indicated concentrations of Nio-MT@S-G MN extracts for 4 h, and cell viability was measured by CCK-8 assay. Data are mean ± SD from three independent experiments. ns, not significant vs. control.

**Figure S4. Quantitative Western blot analysis of ZO-1 protein expression in HaCaT cells.**

HaCaT cells were treated with CTR (PBS), NETs (5 μg/mL), or NETs + Nio-MT@S-G MN (13.7 μM/ml) for 24 h. a) Representative Western blot of ZO-1 and β-Actin (loading control). b) Densitometric quantification of ZO-1 protein levels normalized to β-Actin, expressed as mean ± SD from three independent experiments. *p < 0.05, **p < 0.01; ns, not significant.

**Figure S5. Annexin V-FITC/PI flow cytometry analysis of HaCaT cell apoptosis.**

HaCaT cells were treated with PBS, NETs (5 μg/mL), or NETs + Nio-MT@S-G MN (1.37 μM) for 24 h. The percentages of viable cells (Q4) remained above 97% across all groups, with no notable increase in early (Q3) or late (Q2) apoptotic populations upon NET exposure. Data are representative of three independent experiments, confirming that NETs do not induce apoptosis in HaCaT cells.

**Figure S6. PAD4 inhibition recapitulates the therapeutic effects of Nio-MT@S-G MN in sleep deprivation-aggravated oral ulcers.**

A) Representative macroscopic images of oral ulcers in the OUS, OUS+Cl-amidine, and OUS+Nio-MT@S-G MN groups from day 1 to day 6. Dashed lines indicate the wound margins. B) Representative wound closure trajectories of individual mice in the three groups over time. C) Quantitative analysis of wound healing rates at indicated time points. D) HE staining images showing epithelial morphology and inflammatory infiltration. E) Western blot analysis of CitH3 protein expression in ulcer tissues from the three groups at day 6. β-Actin was used as the loading control. Cl-amidine, a specific PAD4 inhibitor, was administered intraperitoneally at 50 mg/kg daily. Data are presented as mean ± SD. (B) * indicates the difference between the OUS and OUS+Cl-amidine groups, and # indicates the difference between the OUS and OUS + Nio-MT@S-G MN groups.Statistical significance was analyzed as described in the Methods section. *P < 0.05, #P < 0.05, **P < 0.01, ##P < 0.01, ***P < 0.001, ###P < 0.001.

**Figure S7. Component contribution analysis of the Nio-MT@S-G MN system in sleep deprivation-aggravated oral ulcers.**

A) Representative macroscopic images of oral ulcers in the OUS, OUS+Melatonin (free melatonin), OUS+Niosome (blank niosomes), OUS+S-G MN (blank microneedles without niosomes or melatonin), and OUS+Nio-MT@S-G MN (full system) groups from day 1 to day 6. Dashed lines indicate the wound margins. B) Representative wound closure trajectories of individual mice in the five groups over time. C) Quantitative analysis of wound healing rates at indicated time points. Data are presented as mean ± SD. .Statistical significance was analyzed as described in the Methods section. **P < 0.01, ***P < 0.001.

**Table S1. List of abbreviations used in this study.**

| **Abbreviation** | **Full term** |
| --- | --- |
| Biological terms | |
| NETs | Neutrophil extracellular traps |
| PAD4 | Peptidylarginine deiminase 4 |
| CitH3 | Citrullinated histone H3 |
| MPO | Myeloperoxidase |
| NE | Neutrophil elastase |
| IL-1β | Interleukin-1β |
| TNF-α | Tumor necrosis factor-α |
| ZO-1 | Zonula occludens-1 |
| Experimental groups | |
| SD | Sleep deprivation |
| OU | Oral ulcer (normal sleep) |
| OUS | Oral ulcer with sleep deprivation |
| Materials & formulations | |
| SilMA | Silk fibroin methacrylate |
| GelMA | Gelatin methacryloyl |
| Nio-MT@S-G MN | Melatonin-loaded niosome-incorporated SilMA/GelMA microneedle |
| BN | Blank niosome |
| MN | Melatonin niosome |
| Nio-MT | Melatonin-loaded niosome |
| S-G MN | Blank SilMA/GelMA microneedle |
| Nio@S-G MN | Blank niosome-loaded SilMA/GelMA microneedle |
| Characterization & assays | |
| TEM | Transmission electron microscopy |
| SEM | Scanning electron microscopy |
| FTIR | Fourier transform infrared spectroscopy |
| CCK-8 | Cell Counting Kit-8 |
| RT-qPCR | Reverse transcription‑quantitative real‑time PCR |
| Other | |
| DMSO | Dimethyl sulfoxide |
| FBS | Fetal bovine serum |
| EER | Encapsulation efficiency rate |
| DLR | Drug loading rate |

Abbreviations are defined at their first appearance in the text. This table provides a quick reference for the abbreviations used throughout the manuscript, grouped by category for clarity.

**Table S2. Fluorescence standard curve of melatonin.**

FI

| concentration of Melatonin(μg/ml) | FI |
| --- | --- |
| 0.4 | 1.125 |
| 0.8 | 1.723 |
| 1.2 | 2.16 |
| 2 | 3.407 |
| 2.4 | 3.615 |
| 2.8 | 4.123 |
| 3.2 | 4.443 |
| 3.6 | 5.185 |
| 4 | 5.297 |

Melatonin standard solutions at different concentrations were prepared in methanol and analyzed by fluorescence measurement. Fluorescence intensity (FI) was recorded at an excitation wavelength of 285 nm and an emission wavelength of 345 nm. The resulting calibration curve showed a good linear relationship between melatonin concentration and fluorescence intensity (y = 1.1784x + 0.7822, R² = 0.9906), and was used for the determination of melatonin encapsulation efficiency, drug loading, and in vitro release.

**Table S3. Sequences of the primers used in this study.**

| GENES | | FORWARD PRIMER SEQUENCE (5’-3’) | REVERSE PRIMER SEQUENCE (5’-3’) |
| --- | --- | --- | --- |
| Il-1β | CACCTCTCAAGCAGAGCACAG | | GGGTTCCATGGTGAAGTCAAC |
| Tnf-α | CCAAATGGCCTCCCTCTCAT | | TGGTGGTTTGCTACGACGTG |
| PADi4 | CGTGAGGCTCTTCTCCGATT | | GCTCTCCACATAGGCATTCTG |
| Elane | GGATCTTCGAGAATGGCTTTG | | GCACGTTGGCGTTAATGGT |
| ZO-1 | GTTGTCCCTCCTCTGATACCTTC | | TGGCAGTGTCATTCACATCTTTC |
| β-Actin | AGAAAATCTGGCACCACACCT | | GATAGCACAGCCTGGATAGCA |
